# European Guideline on Pre‐Operative Prevention of Surgical Site Infections Following Digestive Surgery: A Joint Update of the WHO SSI Guideline for Gastrointestinal Surgery by UEG, ESCP, EAES, and SIS‐E

**DOI:** 10.1002/ueg2.70128

**Published:** 2025-10-25

**Authors:** UEG/ESCP/EAES/SSI‐E SSI Prevention Working Group, Adele Sayers

**Affiliations:** ^1^ Sheffield Teaching Hospitals Sheffield UK; ^2^ University of Sheffield Sheffield UK

## Abstract

This is an update of the WHO SSI guideline published in 2018, focussing on areas pertinent for gastrointestinal (GI) surgery and hepatobiliary and pancreatic (HBP) surgical procedures. Based on new information and appraisal of current evidence, the following recommendations can be suggested: During skin preparation, we suggest using alcohol‐based chlorhexidine for clean, clean‐contaminated, and contaminated GI and HBP surgical field preparation in the absence of a mucous membrane (e.g., stoma, genitalia, anus). Preoperatively, we suggest that corticosteroids and anti‐TNF medication be discontinued. No recommendations could be made on the following comparisons, given the paucity of high‐quality evidence and panel discussion not favouring one intervention over the other based on clinical experience: 2%–3% chlorhexidine gluconate versus aqueous povidone‐iodine for surgical field preparation. 4%–5% chlorhexidine gluconate versus aqueous povidone‐iodine for surgical field preparation. Aqueous chlorhexidine gluconate versus aqueous povidone‐iodine for surgical field preparation Pre‐operative dexamethasone in single‐dose versus no pre‐operative dexamethasone for patients undergoing GI surgery. Discontinuing Vedolizumab preoperatively versus continuing Vedolizumab preoperatively for patients undergoing GI surgery. Discontinuing Ustekinumab preoperatively versus continuing Ustekinumab preoperatively for patients undergoing GI surgery.

## Graphical abstract

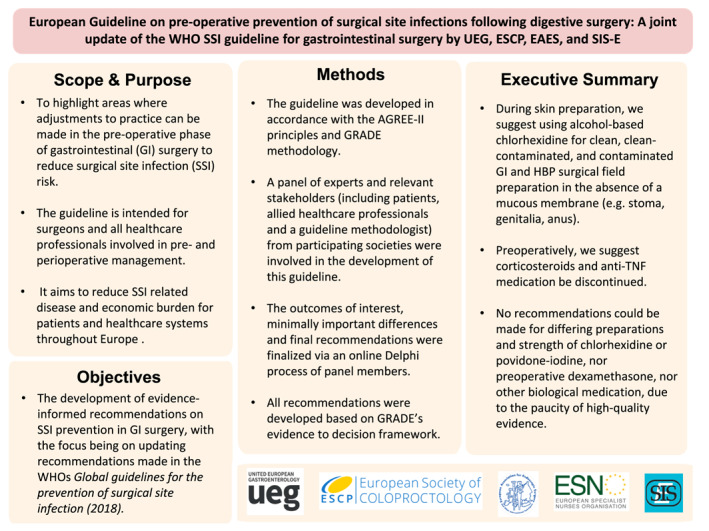

## 1 Lay Summary

Surgical site infection (SSI) is a complication that occurs after a surgical operation [1]. The occurrence is reported to be about one in eight, which could result in a prolonged hospital stay and has a significant impact on patient's quality of life [2, 3].

The World Health Organization (WHO) published their updated international SSI guidelines in 2018 [1]. This included evidence available at the time relating to all types of surgery. Since then, new evidence has emerged and we feel it is important to update the guideline, particularly in relation to gastrointestinal surgery as this is a type of surgery that could expose patients to gut content and thus could result in a higher risk of developing SSIs.

This guideline has specifically focussed on measures before surgical operations that could help to prevent SSIs. We assessed the types of skin preparation (solutions used to clean the skin just before surgery), and whether specific medications need changing or stopping, for example changing types of antibiotics, or stopping medications that can have an impact on the body's ability to fight off infection.

Our recommendations are

- During skin preparation, we suggest using alcohol‐based chlorhexidine in the absence of a mucous membrane (e.g., stoma, genitalia, anus).
- We suggest that corticosteroids and anti‐TNF medication (medication i.e. used to decrease inflammation and treat conditions such as inflammatory bowel disease) be stopped before surgery.

## 2 Scope and Purpose

A surgical site infection (SSI) is a postoperative infection that occurs specifically in a body region where surgery has taken place [1]. SSIs are an important clinical problem, especially in surgical procedures on the digestive tract, which naturally harbours multiple bacteria.

SSIs have a significant impact on patients, as a prolonged stay in hospital and the subsequent complications related to SSI occurrence significantly affect patients' well‐being and quality of life [2, 3].

The development of SSIs (across all surgical specialities) has been reported to cost between €12,167 and €32,000 per patient [4]. European data from 2004 suggested that SSIs were under‐reported, with their impact on economic costs 20 years ago ranging from 1.47 to 19.1 billion Euro per year [5, 6]. The worldwide risk of developing an SSI after GI surgery is 12.3%, the risk of which can increase to nearly 40%, if undergoing contaminated (i.e., infection already present) surgery in a low‐income country [7]. SSIs are therefore a substantial financial burden on healthcare systems worldwide.

SSIs can account for over 36% of all hospital acquired infections and are frequently associated with multidrug‐resistant bacteria [8]. Drug‐resistant infection can be as high as 45% for *Staphylococcus aureus* and 15% for *Escherichia coli* [9]. The incidence of multidrug resistant SSIs is rising, which will place further burden on patients and healthcare systems in the future.

The purpose of this guideline is to highlight areas where adjustments can be made in practice to reduce the risk of SSI during the pre‐operative phase for patients undergoing gastrointestinal surgery. The guideline is intended not only for surgeons but also for healthcare professionals involved in patient preparation and intraoperative management. It aims to reduce SSI related disease and economic burden for patients and healthcare systems throughout Europe.

## 3 Objectives

This guideline aimed to develop evidence‐informed recommendations on the prevention of SSI in gastrointestinal surgery; to reduce the incidence of SSIs, improve patient care and experience, and reduce the significant economic burden that SSIs have in health systems across Europe. We focussed on updating recommendations made in the WHO’s ‘Global guidelines for the prevention of surgical site infection’ updated in 2018 [1]. We chose to specifically focus on gastrointestinal surgery and the pre‐operative phase as to date there are no high‐quality guidelines specifically focussing on this subject matter.

## 4 Methods

### 4.1 Research Questions

The PICO (population, intervention, comparator, and outcome) framework was used to address the effectiveness and the safety of an intervention to prevent SSIs.

All research questions from the 2018 WHO Guidelines to prevent SSI were evaluated to determine if any new evidence was available from 2018 to the present day [1].

The topics from the WHO guideline where new evidence was available were types of surgical site preparation, antibiotic prophylaxis for Extended‐spectrum beta‐lactamase (ESBL)‐producing bacteria, and pre‐operative immunosuppressive agents. The research questions and their corresponding PICOs can be found in the protocol (Supporting Information S2: Appendix 2). There was also new evidence for the topic of mechanical bowel preparation, but this is not included in this guideline, as this topic was recently covered in EAES's rapid guideline ‘EAES, SAGES, and ESCP rapid guideline: bowel preparation for minimally invasive colorectal resection’ [10].

### 4.2 Target Population

This guideline targets adult patients undergoing gastrointestinal surgery (including hepatobiliary and pancreatic surgery) in Europe. We did not have any restriction regarding existing comorbidities and/or type of disease (benign vs. malignant for example).

### 4.3 Target Users, Preferences and Views

The target users of this guideline are surgeons, anaesthetists, infectious diseases specialists, microbiologists, hospital management, pharmacists, nurses, and general practitioners involved in the care of patients who undergo digestive surgery. Patients' views were sought from patient representatives with regard to interventions and the weighing of benefits against risks.

### 4.4 Methodological Support

All members of the guideline steering committee are current or past members of the European Society of Coloproctology's (ESCP) guideline committee and a guideline methodologist was involved throughout development.

### 4.5 Stakeholder Involvement

This was an international and multi‐society collaboration of the European Society of Coloproctology (ESCP), Surgical Infectious Society Europe (SIS‐E), European Association of Endoscopic Surgery (EAES), and United European Gastroenterology (UEG) to provide up to date guidance on the prevention of SSIs.

A panel of experts from the participating societies was established as a steering group. The guideline development panel (GDP) was selected from members of the four European Societies and included relevant stakeholders (with patient and nursing representatives). GDP was balanced according to gender, age, expertise, and geographical location. Patients were invited to join the preliminary meetings, to provide feedback on the protocol which was developed a priori, to vote on choosing relevant outcomes and to vote on the final recommendations. Involved patients were asked for their feedback on the manuscripts and were asked to assist in the writing of a lay summary intended for patients once the guidelines were completed.

### 4.6 Rating the Importance of Outcomes

In line with the GRADE methodology, the importance of outcomes was voted on by the guideline panel [11]. When voting on the importance, the GRADE scale was used. This scale scores outcomes of lower importance 1–3, important outcomes 4–6 and outcomes deemed critical 7–9. After online voting by the guideline panel, the steering group ranked each outcome into three categories: not important, important, and critical. Prior to voting, it was decided that a maximum of six outcomes would be used for this guideline, in line with GRADE recommendations for seven or fewer outcomes per guideline [12].

The outcomes deemed critical were:

— Overall SSI rate
— Superficial SSI rate
— Deep SSI rate
— Organ space SSI rate
— Overall mortality

The sixth critical outcome, quality of life, was included with the aforementioned outcomes for formulation of the PICOs. However, as none of the included studies reported quality of life as an outcome measure, we have not been able to include it as an outcome when formulating recommendations.

### 4.7 Setting the Minimally Important Differences

Minimally important differences (MID) can be developed based on anchor‐based, distribution‐based or consensus‐based methodologies [13]. The latter was used within this guideline to set the MID, as given the limited reporting of SSIs according to the Centre for Disease Control classification, it was deemed most appropriate [14].

An anonymous survey of guideline panel members was conducted to define what would be classed as the minimal important difference for outcomes between the interventions per 1000 patients. The minimally important difference was defined as the smallest difference in score in the outcome of interest that would lead to the panel member to choose one intervention over the other [12]. The median of the minimal important difference was used for each outcome. If a significant discrepancy was found in the MID between panel members, then consensus would be reached via a Delphi exercise; however, no significant discrepancy was found for the outcomes included in the guideline. The minimal important differences for each outcome were:

— Overall SSI rate: 25 per 1000 patients fewer/more
— Superficial SSI rate: 25 per 1000 patients fewer/more
— Deep SSI rate: 10 per 1000 patients fewer/more
— Organ space SSI rate: 10 per 1000 patients fewer/more
— Mortality rate: 5 per 1000 patients fewer/more

### 4.8 Search Methods

According to PICO, PubMed/Medline, EMBASE, Cochrane Central database, Trip (for grey literature) and CINAHL were interrogated with no language restrictions to identify systematic literature reviews. Identified systematic reviews were assessed by AMSTAR 2 [15]. If no relevant or high‐quality systematic reviews were available, a new systematic review was conducted. Older but relevant literature was included if deemed necessary. The literature search was performed with the assistance of clinical information specialists (www.ksrevidence.com) and full search strategies can be found in Supporting Information S1: Appendix 1.

Given that the last update of systematic literature during the development of WHO SSI guidelines was April 2018, literature published since May 2018 was the main focus.

### 4.9 Study Selection and Evidence Selection Criteria

All studies were independently reviewed by two members of the steering group (AS, YM) in accordance with Cochrane methodology [16]. Any disagreements in final study selection between the two reviewers were discussed with the rest of the steering group until consensus was reached.

If no high‐quality systematic reviews were available, then new systematic reviews and meta‐analyses were performed by the steering group in accordance with Cochrane standards. Evidence was primarily collected from randomised controlled trials. For RCTs, the Cochrane risk of bias tool (RoB2) was used, including the RoB2 specifically for cluster‐trials where appropriate. If there was no RCT, data from observational studies were synthesised if deemed suitable following ROBINS‐I assessment and exclusion of studies at critical risk of bias (uncontrolled confounders).

The full protocol, which includes all PICOs and the inclusion and exclusion criteria, can be found in Supporting Information S2: Appendix 2.

### 4.10 Strengths and Limitations of Evidence

Evidence was assessed according to GRADE methodology, according to the framework of assessing study design (risk of bias), inconsistency, imprecision, indirectness, and publication bias [12]. Data were collated using GRADEPro [17]. The quality of the evidence was summarised using the GRADE approach and classified into four categories: ‘high’, ‘moderate’, ‘low’, and ‘very low’.

### 4.11 Evidence to Decision Framework and Developing Recommendations

The guideline panel reviewed all evidence tables and provided judgement on the magnitude of benefit/harm, certainty of evidence and the implicated costs, equity, acceptability, and feasibility of each intervention. Panel members were invited to take part in an online Delphi process to discuss and formulate recommendations prior to being invited to propose modifications to the draft recommendations then developed by the steering committee. All recommendations drafted by the steering committee were then reviewed and agreed upon by the guideline panel.

### 4.12 Wording of Recommendations

Wording considered for recommendations was done as per the GRADE handbook, depending on the strength of the recommendation:

— Strong recommendation: ‘*The panel recommend*’
— Weak/conditional recommendation: ‘*The panel suggest*’

As per the GRADE handbook ‘strong recommendations imply that most individuals will be best served by the recommendation’, whereas for a weak or conditional recommendation ‘there is a need to consider more carefully the patients' individual circumstances, preferences and values’. Practically, the decision to follow weak or conditional recommendations may change depending on patient values, resources available, or the patient setting [12].

## 5 Results

### 5.1 Interventions and Comparisons

In total, 12 different interventions and comparisons were highlighted for the three different research questions: (1) what skin preparation should be used for GI and HBP surgery to decrease SSI incidence? (2) Should immunosuppressive medication be stopped prior to GI and HBP surgery to decrease SSI incidence? (3) Should Abx prophylaxis for GI and HBP surgery be changed in high‐ESBL bacteria prevalent areas or among those that are carriers? The following interventions and comparisons, in conjunction with the five critical outcomes, were used to formulate the PICOs (Supporting Information S2: Appendix 2).

Q1. Alcohol‐based chlorhexidine gluconate compared to aqueous povidone‐iodine.

Q2. Aqueous chlorhexidine gluconate compared to aqueous povidone‐iodine.

Q3. Alcohol‐based chlorhexidine gluconate 4%–5% compared to aqueous povidone‐iodine.

Q4. Alcohol‐based chlorhexidine gluconate 2%–3% compared to aqueous povidone‐iodine.

Q5. Alcohol‐based chlorhexidine gluconate 2%–3% compared to alcohol‐based povidone‐iodine.

Q6. Preoperative dexamethasone in single‐dose versus no pre‐operative dexamethasone.

Q7. Discontinuation compared to continuation of corticosteroids.

Q8. Discontinuation compared to continuation of anti‐TNF.

Q9. Discontinuation compared to continuation of Vedolizumab.

Q10. Discontinuation compared to continuation of Ustekinumab.

Q11. Change compared to no change in antibiotic prophylaxis in areas with high (> 10%) ESBL‐producing Enterobacteriaceae prevalence.

Q12. Modification compared to no modification of antibiotic prophylaxis in patients who are known carriers of ESBL‐producing Enterobacteriaceae.

### 5.2 Search Results

There were no high‐quality systematic reviews available for any of the included research questions. Therefore, new systematic reviews were conducted. We identified 76 studies that fit the predetermined selection criteria for the guidelines. The PRISMA flowcharts for each PICO can be found in Supporting Information S3: Appendix 3. All studies focussing on skin preparation (Q1‐5) were randomised controlled trials, as were all those investigating the effect of dexamethasone (Q6) on SSI rates. All other studies identified were observational studies (Q7–11).

### 5.3 Subgroup Analysis

Subgroup analysis could not be performed to inform procedure‐specific or contamination‐specific recommendations due to the paucity of data and resultant risk of significant imprecision.

### 5.4 Formulation of Recommendations

Recommendations were developed based on GRADE's Evidence to Decision framework, which addressed the quality of evidence, balance between risks and benefits, implementability, use of resources (cost), equity and feasibility, acceptability, and patients' values and preferences.

### 5.5 Recommendations

All recommendations are **conditional**.

For patients undergoing gastrointestinal, hepatobiliary and pancreatic procedures.

- The panel suggests the use of alcohol‐based chlorhexidine gluconate [very low certainty of evidence]
- The panel suggests that corticosteroid medication should be discontinued pre‐operatively [very low certainty of evidence]
- The panel suggests anti‐TNF medication should be discontinued pre‐operatively [very low certainty of evidence]

No recommendations could be made for the following comparisons:

- Aqueous chlorhexidine gluconate compared to aqueous povidone‐iodine
- Alcohol‐based chlorhexidine gluconate 4%–5% compared to aqueous povidone‐iodine
- Alcohol‐based chlorhexidine gluconate 2%–3% compared to aqueous povidone‐iodine
- Alcohol‐based chlorhexidine gluconate 2%–3% compared to alcohol povidone‐iodine
- Pre‐operative single‐dose dexamethasone compared with no dexamethasone
- Discontinuation compared to continuation of Vedolizumab
- Discontinuation compared to continuation of Ustekinumab
- Change compared to no change in antibiotic prophylaxis in areas with high (> 10%) ESBL‐producing Enterobacteriaceae prevalence
- Modification compared to no modification of antibiotic prophylaxis in patients who are known carriers of ESBL‐producing Enterobacteriaceae

### 5.6 Justifications

#### 5.6.1 Considerations of Benefits and Harms

Both the benefits and the risks of all interventions were evaluated. The potential trade‐off between the benefits of all interventions on SSI prevention and the potential harms were carefully considered.

#### 5.6.2 Link Between Recommendations and Evidence

In addition to the GRADE Evidence to Decision Framework, thought processes by the guideline members leading to recommendations were made explicit with detailed descriptions.

A summary of the evidence to decision (EtD) tables can be found in Table 1.

**TABLE 1:** Evidence to decision table.

	Comparison	Desirable effect	Meeting minimally important difference?	Undesirable effect	Certainty of evidence	Additional consideration
Q1	Alcohol‐based chlorhexidine gluconate (CHG) compared to aqueous povidone‐iodine (PVI)	There will be **47 fewer SSIs per 1000 patients** with the use of alcohol‐based chlorhexidine (all concentrations)	Yes (MID = 25 in 1000)	There will be **1 more adverse event** (skin irritation/skin reactions) per 1000 patients if using aqueous PVI	Very low	None of the studies reported on mortality, overall survival or complications, which were deemed critical outcome by the panel. There is no reliable data on the different classifications of SSIs ‐ results reported were overall SSIs only. Certainty was downgraded to very low as very serious indirectness to the research question was found. Additionally, some studies included non‐GI or HPB abdominal surgeries, the data for which could not be isolated within study results [18, 19, 20, 21, 22, 23, 24, 25, 26]
Q2	Aqueous chlorhexidine gluconate compared to aqueous povidone‐iodine	There will be **4 fewer SSIs per 1000 patients** with the use of aqueous chlorhexidine	No (MID = 25 in 1000)	This RCT did report on adverse skin reactions, and none were experienced in the aqueous CGH nor aqueous PVI group	Very low	The data within the single RCT is only for clean‐contaminated surgery and it was not clear if it was being used in preference to alcohol‐based solutions, due to the presence of a mucous membrane. Each group had a different pre‐operative washing solution prior to application of the skin preparation, which lowered precision of the results. Meta‐analysis results were noted to have wide 95% confidence intervals which additionally included 1 [27]
Q3	Alcohol‐based chlorhexidine gluconate 4%–5% compared to aqueous povidone‐iodine	There will be **20 fewer SSIs per 1000 patients with** the use of chlorhexidine gluconate 4%–5% concentration	No (MID = 25 in 1000)	There would be **6 more adverse reactions** per 1000 patients if using aqueous PVI	Very low	There was serious bias in inconsistency, indirectness, and imprecision. This was due to the RCTs not specifying the type of surgery (including all abdominal and non‐abdominal surgery), including all clean/clean‐contaminated/contaminated surgery and one RCT using different pre‐operative skin washing solutions prior to surgical skin preparation. One of the studies included patients undergoing gynaecological surgery, some of whom also underwent GI surgery at the same time, while the other study did include GI surgery along with other non‐specified specialities (e.g. vascular, breast). Meta‐analysis results were noted to have wide 95% confidence intervals which additionally included 1 [22, 24]
Q4	Alcohol‐based chlorhexidine gluconate: 2%–3% compared to aqueous povidone‐iodine	There will be **52 fewer SSIs per 1000 patients with** the use of chlorhexidine gluconate 2%–3% (alcohol)	Yes (MID = 25 in 1000)	There was 0 fewer adverse event in the 2%–3% alcohol CHG group	Very low	Two of the five RCTs had a high risk of bias due to inadequate randomisation or missing outcome data, and two of the RCTs did not report on adverse events. There was a mixture of clean, clean‐contaminated, and contaminated surgeries within the RCTs, with no subgroup data available. Meta‐analysis results were noted to have wide 95% confidence intervals which additionally included 1 [18, 20, 21, 23, 25]
				Numbers were very small across all groups		
Q5	Alcohol‐based chlorhexidine gluconate 2%–3% compared to alcohol povidone‐iodine	There will be **7 fewer superficial SSIs per 1000 patients** if 2%–3% chlorhexidine gluconate (CHG) is used	No (MID = 25 in 1000)	There were **18 more organ space SSIs** in the 2%–3% CHG group when compared to the alcohol PVI, which was deemed clinically significant by the panel (MID = 10 in 1000). There were 11 more SSIs in the 2%–3% CHG, which was deemed not significant (MID = 25 in 1000)	Very low	There was serious indirectness (one RCT included GI and cardiac procedures, while one of the RCTs recorded multiple classification of SSIs for single patients). There was bias due to occasional deviation from protocol in one RCT. Meta‐analysis results were noted to have wide 95% confidence intervals which additionally included 1 [28, 29]
Q6	Preoperative single‐dose dexamethasone compared to no dexamethasone	There will be **3 fewer SSIs per 1000 patients** and **25 fewer superficial SSIs per 1000 patients** if no preoperative dexamethasone is administered in single‐dose	Yes, for superficial SSI (MID 25 in 1000)	There would be **14 more organ space SSI per 1000** (MID = 10 in 1000) patients if no preoperative dexamethasone was given in a single‐dose	Very low	There was high risk of bias in the included studies. Comparative groups were heterogeneous, with dexamethasone versus placebo in most studies, but also dexamethasone given during surgery versus no treatment or another anti‐emetic or analgesic drug in the control group (both blinded and non‐blinded). Most of the included studies used an intermediate dose of dexamethasone (8–10 mg). There was 1 study that used high dose dexamethasone (> 12 mg) which showed a negative effect with stopping dexamethasone (increase in SSI and mortality rates). Meta‐analysis results were noted to have wide 95% confidence intervals which additionally included 1 [30, 31, 32, 33, 34, 35, 36, 37, 38, 39, 40, 41]
Q7	Discontinuation compared continue corticosteroids	There will be **21 fewer SSIs per 1000 patients**, **20 fewer organ space SSIs per 1000 patients,** and **1 fewer death per 1000 patients** if corticosteroids are discontinued preoperatively.	Yes, for organ space SSI (MID = 10 in 1000) but not for overall SSI (MID = 25 in 1000) or mortality (MID = 5 in 1000).	There will be **32 per 1000 more superficial SSIs** if corticosteroids are discontinued preoperatively.	Very low	For overall SSIs, there were a wide variety of included surgical procedures, preoperative medications, heterogeneous baseline diagnoses (Crohn's, UC, cancers etc.), dose of corticosteroids and timing of corticosteroid cessation (imprecision = very serious). Certainty was deemed to be very low for different SSI classification and mortality due to missing adjustments for confounders, high heterogeneity and wide 95% CI intervals. Furthermore, very few studies reported on classification of SSI and mortality [42, 43, 44, 45, 46, 47, 48, 49, 50, 51, 52, 53, 54, 55, 56, 57]
				This did meet the minimally important clinical difference. (MID = 25 in 1000)		
Q8	Discontinuation compared continue anti‐TNF	There will be **35 fewer SSIs per 1000 patients** and **10 fewer superficial SSIs per 1000 patient**s if anti‐TNF medication is discontinued pre‐operatively	No for overall SSI and superficial SSI. Yes, for organ space SSI (MID = 25 in 1000)	There will be **4 more deep SSIs per 1000** if anti‐TNF medication is *discontinued* pre‐operatively. (MID = 10 in 1000)	Very low	High heterogeneity among studies due to different definition of outcomes (SSI, intra‐abdominal sepsis, overall complications), many confounders (different pre‐operative exposure to steroids, different rate of primary anastomosis, different rate of surgical approaches), different timing of suspension before surgery, and different timing for outcomes [44, 46, 47, 50, 51, 53, 58, 59, 60, 61, 62, 63, 64, 65, 66, 67, 68, 69, 70, 71, 72, 73, 74, 75, 76, 77, 78, 79, 80, 81, 82, 83, 84, 85, 86, 87, 88, 89, 90, 91, 92]
		There will be **20 fewer organ space SSIs per 1000 patients** if anti‐TNF medication is discontinued pre‐operatively				
Q9	Discontinuation compared continue Vedolizumab	If vedolizumab were to be discontinued pre‐operatively, there would be **36 fewer total SSIs per 1000 patients**	Yes (MID = 25 in 1000)	There were no documented undesirable effects within the studies	Very low	Data was from 4 small studies only. There was high heterogeneity among the studies and inadequate adjustment for confounders. All studies are observational and retrospective. None of the studies reported on overall complication rates, risk of flares, or patient‐reported outcome measures. Clear classifications of SSIs were not used. There was high heterogeneity among studies, with varying rates of laparoscopic surgery, stoma formation, primary anastomosis, and pre‐operative steroid use. Meta‐analysis results were noted to have wide 95% confidence intervals which additionally included 1 [67, 71, 73, 76]
Q10	Discontinuation compared continue Ustekinumab	There will be **141 fewer SSIs per 1000 patients** and **89 fewer organ space SSIs per 1000 patients** if Ustekinumab is discontinued preoperatively	Both yes (MID = 25 and 10 in 1000, respectively)	There were no documented undesirable effects within the studies	Very low	Data was from two retrospective, observational studies only, with possible overlapping data. Certainty was deemed to be very low for all SSIs due to inadequate control for confounding biases and study heterogeneity. None of the studies reported on overall complication rates, risk of flares, or patient‐reported outcome measures. Clear classifications of SSIs were not used. There was high heterogeneity among studies, with varying rates of laparoscopic surgery, stoma formation, primary anastomosis and pre‐operative steroid use. Meta‐analysis results were noted to have wide 95% confidence intervals which additionally included 1 [76, 93, 94]

Forest plots for each of the comparisons can be found in Supporting Information S4: Appendix 4.

#### 5.6.3 Rationale Behind Recommendations Made

##### 5.6.3.1 Alcohol‐Based Chlorhexidine Gluconate Compared to Aqueous Povidone‐Iodine

Although the quality of evidence was very low, the meta‐analysis showed in favour of the intervention (alcohol‐based CHG) and there was a moderate desirable effect on lowering overall SSI rates, which met the panel members' minimally important difference. The meta‐analysis included all concentrations of CHG and included studies where no concentration had been specified.

None of the included studies reported the SSIs according to the CDC classification (superficial SSI, deep SSI and organ space SSI). With regards to the degree of contamination within the surgery, most studies included clean and clean contaminated surgery, with two studies also including contaminated surgery. Only one of these two studies provided subgroup data. They found a significantly higher rate of SSI for both CHG and PVP‐I in contaminated surgery, but found no significant difference in the rates of SSI in clean contaminated and contaminated surgery between the two different skin preparations.

##### 5.6.3.2 Discontinuation Compared to Continuation of Corticosteroids

The quality of evidence was very low, but the meta‐analysis showed in favour of the intervention (discontinuing corticosteroids) and there was a moderate desirable effect in reducing organ space SSIs with the intervention, which met the panel members' minimally important difference.

The use of corticosteroids in this context refers to pre‐operative corticosteroid use (e.g., prednisolone and hydrocortisone), rather than a single dose of peri‐operative corticosteroid (e.g., dexamethasone). As noted in the previous recommendation, there was a lack of SSI reporting in line with the CDC SSI classification. All studies reported on overall SSI rates, with only 3/16 reporting superficial SSIs. Nearly all (15/16) studies reported organ‐space SSI, with some studies additionally classifying organ‐space SSI in the context of bowel anastomoses as anastomotic leak with varying levels of conservative and operative management. Most studies included patients operated on in the elective and emergency settings, but the source data was not sufficiently separated to allow further analysis of these two groups.

##### 5.6.3.3 Discontinuation Compared to Continuation of Anti‐TNF Medication

There was a large effect noted in decreasing overall SSI and organ space SSI rates with the intervention (stopping anti‐TNF), which met the panel members' minimally important difference. As with previous recommendations, the quality of evidence used to base this recommendation was very low for overall SSI rates and organ space SSI rates, but the meta‐analysis also showed in favour of the recommendation.

All patients within these studies had inflammatory bowel disease and were undergoing bowel resection, strictureplasty, or stoma reversal. As for all studies used within the guideline, for this recommendation there was poor reporting in line with the CDC SSI classification. While all studies reported on the presence of overall SSI, 75% of studies additionally reported organ‐space SSIs, which in the context of bowel anastomoses, as with the previous recommendation, were also reported as anastomotic leaks with varying rates of conservative and operative management. Most studies included patients in the elective and emergency settings, but the source data was not sufficiently separated to allow further analysis of these two groups. In the acute setting, it may not always be possible for patients to undergo a period of optimisation (stopping of anti‐TNF/corticosteroid/nutritional optimisation etc.) prior to surgery. This should be assessed on a patient‐by‐patient basis, depending upon clinical assessment and investigation findings. In these circumstances where a patient cannot undergo a period of optimisation prior to surgery, this recommendation is no longer relevant.

#### 5.6.4 Rationale Behind No Recommendations

##### 5.6.4.1 Aqueous Chlorhexidine Gluconate Compared to Aqueous Povidone‐Iodine

The data for this recommendation came from a single RCT, with significant heterogeneity between the groups, which used different pre‐operative bathing solutions immediately prior to the use of the skin preparation. The quality of the data was deemed to be too poor to be the basis for any recommendations and the small effect noted in the intervention (aqueous CHG) to reduce SSI incidence did not meet the panels’ minimally important difference.

##### 5.6.4.2 Alcohol‐Based Chlorhexidine Gluconate 4%–5% Compared to Aqueous Povidone‐Iodine

The data for this recommendation came from two small RCTs, the certainty of which was deemed to be very low. Each study did not conduct subgroup analyses depending upon the type of surgery (all surgeries, including non‐GI surgery and all classifications of contamination included). Additionally, different pre‐operative bathing solutions were used between the groups in one RCT prior to skin preparation. The quality of the data was deemed to be too poor to be the base for any recommendations, the meta‐analysis did not show in favour of either intervention (alcohol based 4%–5% CGH) nor control and the effect noted in the intervention to reduce SSI incidence did not meet the panels minimally important difference.

##### 5.6.4.3 Alcohol‐Based Chlorhexidine Gluconate 2%–3% Compared to Aqueous Povidone‐Iodine

The data for this recommendation came from five RCTs, two of which were deemed to be at high risk of bias due to inadequate randomisation or missing outcome data. There was a mixture of clean, clean‐contaminated, and contaminated surgeries within the studies, with no subgroup analysis of these different groups. The meta‐analysis did not show in favour of either the intervention (alcohol‐based 2%–3% CHG) or the control. Despite the effect size of the intervention on reducing SSIs meeting the guideline panels’ minimally important difference, the quality of the evidence was deemed to be too low to use as the basis for a recommendation.

##### 5.6.4.4 Alcohol‐Based Chlorhexidine Gluconate 2%–3% Compared to Alcohol‐Based Povidone‐Iodine

While the data for this recommendation came from two moderately sized RCTs, there was serious indirectness in the results, as one RCT included not GI procedures and classified multiple classifications of SSI for single patients with no clear explanation of how they did this. Additionally, one of the RCTs was noted to occasionally deviate away from the protocol. The quality of the data was deemed to be too poor to be the basis for any recommendations, the meta‐analysis did not show in favour of either intervention (alcohol‐based 2%–3% CGH) or control and the effect noted in the intervention to reduce SSI incidence did not meet the panels’ minimally important difference.

##### 5.6.4.5 Discontinuation Compared to Continuation of Ustekinumab

The data for this recommendation came from two moderately sized RCTs within the same unit with possible overlap of data (one of the studies had been excluded from the 2020 Cochrane review for the same reason) [95]. There was a significant heterogeneity between the groups and meta‐analysis did not show in favour of either the intervention (discontinuation of Ustekinumab) or the control at reducing SSIs, despite the effect size on organ‐space infections meeting the panels’ minimally important difference.

##### 5.6.4.6 Discontinuation Compared to Continuation of Vedolizumab

The data for this recommendation came from four small observational studies, all deemed to be of low quality due to their significant heterogeneity and lack of adjustment for significant confounding factors (e.g., concomitant high‐dose steroid use). The quality of the data was deemed to be too poor to be the basis for any recommendations, and the meta‐analysis did not show in favour of either intervention (discontinuation of Vedolizumab) or control; however, the effect noted in the intervention to reduce SSI incidence did meet the panels’ minimally important difference.

##### 5.6.4.7 Change Compared to No Change of Antibiotic Prophylaxis in Areas With High (> 10%) ESBL‐Producing Enterobacteriaceae Prevalence

There were no available studies that met the inclusion criteria specified within the protocol to allow any recommendation to be made.

##### 5.6.4.8 Modification Compared to No Modification of Antibiotic Prophylaxis in Patients Who Are Known Carriers of ESBL‐Producing Enterobacteriaceae

There were no available studies that met the inclusion criteria specified within the protocol to allow any recommendation to be made.

## 6 Discussion

### 6.1 Implications for Policy Makers

— The panel suggests the use of alcohol‐based chlorhexidine gluconate.
  There are no concerns with regards to the use of CHG and health equity across Europe, and no major barriers to the implementation of the use of CHG as skin preparation for clean, clean‐contaminated or contaminated gastrointestinal, hepatobiliary and pancreatic surgical procedures. The slightly higher cost of CHG compared to PVI is off‐set by the significant savings in reducing SSI incidence. Alcohol‐based CHG should be made available in operating theatres to help reduce SSI rates.
— The panel suggests that corticosteroid medication should be discontinued pre‐operatively.
  Given the favourable impact of steroid weaning on postoperative infectious complications, the intervention will likely be acceptable to key stakeholders. Policy makers must ensure that appropriate infrastructure is in place to allow identification of those patients taking corticosteroids pre‐operatively and allow sufficient time for corticosteroids to be weaned or stopped (where possible) prior to surgery. Policy makers should put processes in place to facilitate multidisciplinary working, as patients' corticosteroids are likely initiated or managed by another specialist who should be involved in the discussions about stopping the medication.
— The panel suggests anti‐TNF medication should be discontinued pre‐operatively.
  Policy makers must ensure appropriate infrastructure is in place to allow operative interventions to take place at the nadir of the medication (4‐weeks for Infliximab; so, missing a dose for those on a 4‐weekly regime or between doses for those on an 8‐weekly regime. For Adalimumab, usually a dose is missed, as timing between doses is usually 2‐weeks). There must be infrastructure in place to allow patients to be restarted on their medication 4 weeks post‐operatively if suitable, to prevent multiple missed doses of medication. Policy makers should put processes in place to facilitate multidisciplinary working, as patients' anti‐TNF medication is likely to be initiated or managed by another specialist who should be involved in the discussions about stopping the medication.

### 6.2 Implications for Healthcare Professionals

— The panel suggests the use of alcohol‐based chlorhexidine gluconate.
  While alcohol‐based chlorhexidine gluconate has been shown to decrease overall SSI rates, it is important to remember that it cannot be used in all situations encountered when conducting gastrointestinal surgery. Alcohol‐based preparations cannot be used on a mucous membrane (e.g., intestinal stoma, perineum) or on an open wound. Alternative aqueous preparations must be used for those areas where a mucous membrane or open wound is present. If suitable, aqueous betadine could be used for the mucous membranes as its colouring allows for easy identification of the limit of preparation, with alcohol‐based chlorhexidine being used for the rest of the preparation, up to the limit of the betadine. However, there are no data to suggest that either aqueous chlorhexidine or aqueous betadine is superior to the other, so choice can be surgeons' preference.
— The panel suggests that corticosteroid medication should be discontinued pre‐operatively.
  The application of the recommendation should be contextualised, and a tailored approach should be used considering each patient's individual circumstances and type of surgical procedure. Although the majority of this data involves patients with inflammatory bowel disease (IBD), the recommendation is also likely to apply to patients without IBD who take corticosteroids for other medical conditions. It may be there are concerns in disease recurrence or relapse when steroids are weaned in IBD patients. Therefore, the benefit of controlling the disease with corticosteroids (or alternative medication) against the risk of SSI needs a careful balance. The trade‐off may be variable depending on clinicians' and patients' decisions. The data from this guideline could serve as benchmarks for the risk/benefit evaluation.
— The panel suggests anti‐TNF medication should be discontinued pre‐operatively.
  Taking into consideration the very low certainty of evidence and the need to balance between the potential risk of SSIs and disease flares, the panel suggests, in patients undergoing elective surgery, to plan the operation at the Infliximab nadir, which is 4 weeks after the last infusion (time between doses: 8 weeks). Patients usually recover after 4 weeks, when the next dose is administered, and no treatment interruption occurs. This can both reduce the risk of flares and ensure surgery is distant enough from the last dose to reduce other infectious risks. In patients taking Adalimumab, usually a dose or two is missed as the period between injections is shorter (1 or 2 weeks). It is unclear if this has any effect on disease recurrence, but it is unlikely.

### 6.3 Implications for Patients

Not all outcomes that were important to patients were included in the studies and this was a noted limitation of the studies. While joint‐decision making may not be applicable for all recommendations, due to institutional policy (e.g., skin preparation choice), discussions must be had about discontinuation of medication pre‐operatively. Patients may have concerns with regards to stopping long‐term medications, such as anti‐TNF medication, or may have concerns around postponing surgery (where possible) to allow steroids to be weaned and/or stopped. It is important to discuss the rationale for stopping these medications to help alleviate any concerns the patients may have.

### 6.4 Implications for Researchers

This guideline has highlighted several gaps and limitations in current evidence, which need addressing:

— The need for high‐quality RCTs comparing SSI rates for patients on biologics versus those who have discontinued them, with the timing of discontinuation standardised.
— The need for high‐quality RCTs comparing SSI rates for patients on corticosteroids versus those who have discontinued them, with the timing of discontinuation standardised.
— The need for a better patient stratification (i.e., currently most studies have significant confounders), such as different timings of medication discontinuation; use of other immunosuppressants in conjunction to biologics such as steroids; different rates of restorative and non‐restorative operations across groups (anastomosis vs. stoma formation), different rates of minimally invasive and open approaches across groups, etc.
— The need for additional outcome measures for all studies, mainly patient‐reported outcome measures (such as quality of life), the incidence of any adverse events (e.g. disease flares off biological medication) and the incidence of different SSI classes (e.g., superficial, deep, organ space), which was missing in most studies.

For those topics we could not formulate recommendations, until new high‐quality evidence is available, any recommendations formulated will need to follow consensus statement methodology, in line with ACCORD reporting standards [96].

### 6.5 Barriers and Facilitators

Multicomponent strategies involving a combination of research (dissemination through email newsletters and social media), ability (providing users with additional resources or tools, such as a visual abstract) and motivational strategies (presentations by key opinion leaders) will be used for dissemination. This approach is likely to encourage implementation as it is more effective than using one strategy alone, particularly for guideline adherence.

### 6.6 Implementation and Dissemination

We will consider producing a snapshot or visual abstract as supplementary material for dissemination and linking to algorithms where applicable on the UEG website. The guidelines will also be uploaded to the UEG Guideline App.

The guideline will be published in UEGJ as an open access article and will be published in any other journal that allows publication of the abbreviated version of the guideline. The full guideline will be made available on the websites of ESCP, SIS‐E, EAES and UEG via a link to the UEG‐website. Evidence‐based prevention and management pathways are summarised in flow algorithms that will inform the development of a UEG smartphone application.

A dedicated communication strategy was developed in collaboration with the Communication Committee of ESCP and the participating societies.

Dedicated educational events and activities (congresses, webinars, courses, interviews) with continued medical education (CME) credits have been organised, with the recommendations presented at the ESCPs annual congress. Emails, newsletters, social media content and presentations by key opinion leaders were used for wider dissemination.

### 6.7 Monitoring/Auditing Criteria

Local projects can be used to assess implementation of the guideline, and to identify any facilitators/barriers to the use of this guideline. We recommend an adherence of 80% to interventions.

### 6.8 Updating Procedure

As new evidence emerged within 5 years from the conception of the WHO SSI guideline, review of evidence and update of this guideline were considered at 5 years from final guideline publication. If little or no new evidence is available at 5‐years, this time interval may be extended.

## Ethics Statement

The authors have nothing to report.

## Consent

The authors have nothing to report.

## Conflicts of Interest

Yasuko Maeda is the current guideline editor of UEGJ. The rest of the submitting group, including the guideline group, does not have any conflicts of interest to declare.

## Supporting information


Supporting Information S1

Supporting Information S2

Supporting Information S3

Supporting Information S4

## Data Availability

The authors have nothing to report.
