# Supplementary material for: European Guideline on Pre‐Operative Prevention of Surgical Site Infections Following Digestive Surgery: A Joint Update of the WHO SSI Guideline for Gastrointestinal Surgery by UEG, ESCP, EAES, and SIS‐E
Source: United European Gastroenterol J. 2025 Oct 25;13(10):1887–904. doi: 10.1002/ueg2.70128 (PMC12704574; doi:10.1002/ueg2.70128)
Supplement: Supplementary file 1 — Supporting Information S1 [file UEG2-13-1887-s003.docx]

# Appendix 1: Search strategy

**Embase/Medline**

*SSIs*

1 *surgical infection/

2 ((wound$ or site$) adj4 infect$ adj4 (surg$ or post-operat$ or postoperat$ or preoperat$ or pre-operat$ or perioperat$ or peri-operat$)).ti,ab,ot.

3 ((surg$ or post-operat$ or postoperat$ or preoperat$ or pre-operat$ or perioperat$ or peri-operat$) adj4 (wound$ or site$) adj4 infect$).ti,ab,ot.

4 (SSI or SSIs).ti,ab,ot.

5 1 or 2 or 3 or 4

*GI Surgery*

6 exp *gastrointestinal surgery/

7 exp *biliary tract surgery/

8 exp *abdominal surgery/

9 (("alimentary canal$" or "alimentary tract$" or "digestive canal$" or "digestive tract$" or "biliary tract" or hepatobiliary or hepato biliary or "intestinal canal$" or gastric or gastroduodenal or gastro duodenal or gastrointestin$ or intestin$ or GI or bariatric or stomach or pancreas or pancreat$ or digestive or bowel or colon or rectum or colorectal or colo-rectal or anus or anal or appendix or hemorrhoid$ or haemorrhoid$ or duodenum or jejunum or ileum) adj3 (surg$ or resection$ or operat$ or bypass or papillotomy or papillectomy)).ti,ab,ot.

10 (anoplasty or fundoplication).ti,ab,ot.

11 (gastric band$ or gastrectomy or weight loss surgery).ti,ab,ot.

12 (gastroduodenostomy or gastro duodenostomy or gastroenterostomy or gastro-enterostomy or gastrojejunostomy or gastro jejunostomy or gastropexy or gastroplasty).ti,ab,ot.

13 (ampullectomy or appendectomy or polypectomy or enterostomy or hepatectomy or oesophagectomy or esophagectomy or colectomy or pancreaticoduodenectomy or pancreatico-duodenectomy).ti,ab,ot.

14 (c?ecostomy or colostomy or duodenostomy or ileostomy or jejunostomy).ti,ab,ot.

15 (abdominoperineal excision or abdomino-perineal excision or pelvic exenteration or rectal extirpation).ti,ab,ot.

16 (vagotomy or vagus denervation or vagus nerve resection or vagus resection).ti,ab,ot.

17 (pyloroplasty or pylorus ligation).ti,ab,ot.

18 or/6-17

19 5 and 18

20 limit 19 to yr="2014 -Current"

*RCT and Date Limit*

21 crossover-procedure/ or double-blind procedure/ or randomized controlled trial/ or single-blind procedure/

22 (random$ or factorial$ or crossover$ or cross over$ or cross-over$ or placebo$ or (doubl$ adj blind$) or (singl$ adj blind$) or assign$ or allocat$ or volunteer$).ti,ab,ot.

23 21 or 22

*Exclusion of animal-only studies*

24 animal/ or animal experiment/

25 (rat or rats or mouse or mice or murine or rodent or rodents or hamster or hamsters or pig or pigs or porcine or rabbit or rabbits or animal or animals or dogs or dog or cats or cow or bovine or sheep or ovine or monkey or monkeys).ti,ab,ot,hw.

26 24 or 25

27 exp human/ or human experiment/

28 26 not (26 and 27)

29 23 not 28

**30 20 and 29**

**KEY**

| Exp *gastrointestinal surgery/ | 'exploded' subject heading (searches all indexing terms below it in the thesaurus hierarchy). * indicates that the search is looking only for 'focused' subject headings (i.e. the most relevant) |
| --- | --- |
| $ | Truncation |
| ? | Embedded truncation (allows for spelling variations) |
| Adj4 | within 4 words of … |
| Adj3 | within 3 words of … |
| .ti,ab,ot,hw. | Searches Title, Abstract and Original Title and Heading Word fields |
