# Supplementary material for: European Guideline on Pre‐Operative Prevention of Surgical Site Infections Following Digestive Surgery: A Joint Update of the WHO SSI Guideline for Gastrointestinal Surgery by UEG, ESCP, EAES, and SIS‐E
Source: United European Gastroenterol J. 2025 Oct 25;13(10):1887–904. doi: 10.1002/ueg2.70128 (PMC12704574; doi:10.1002/ueg2.70128)
Supplement: Supplementary file 2 — Supporting Information S2 [file UEG2-13-1887-s004.docx]

**Appendix 2: Protocol: European Guideline on pre-operative prevention of surgical site infections following digestive surgery – a joint update of the WHO SSI guideline by UEG, ESCP, EAES, and SIS-E**


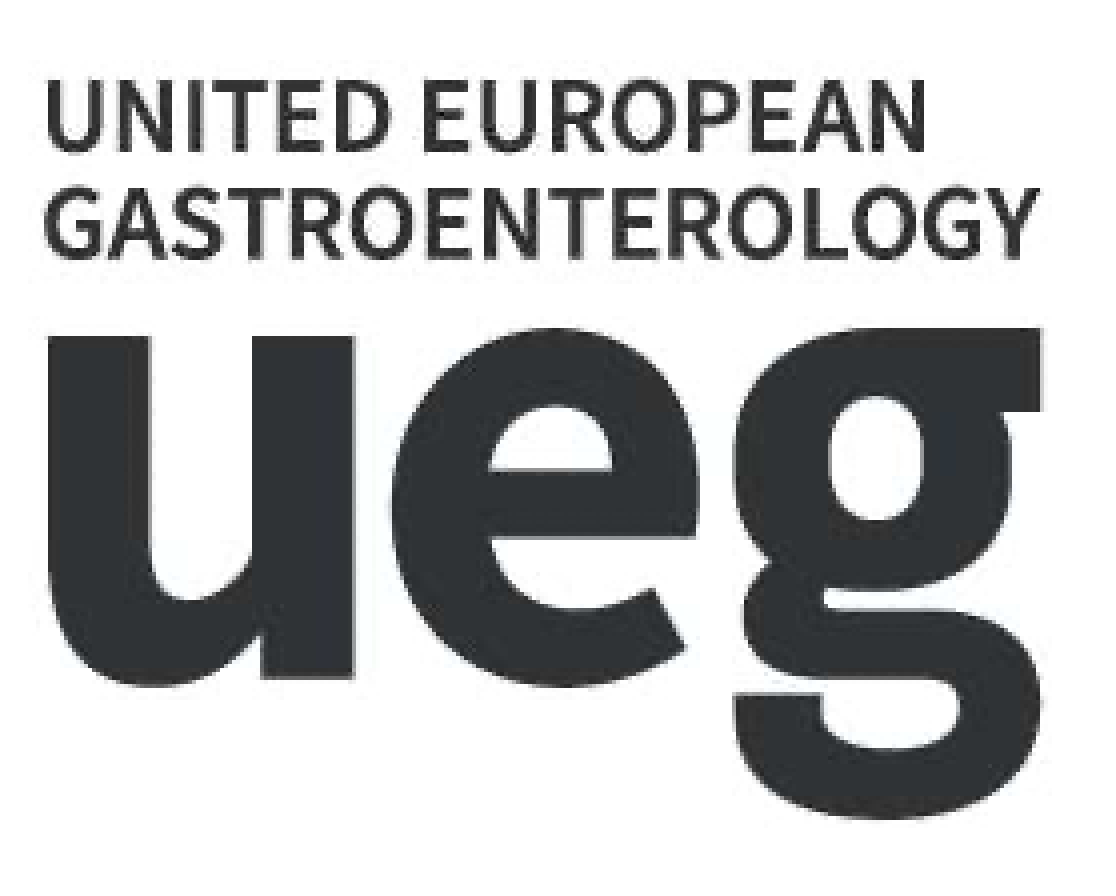

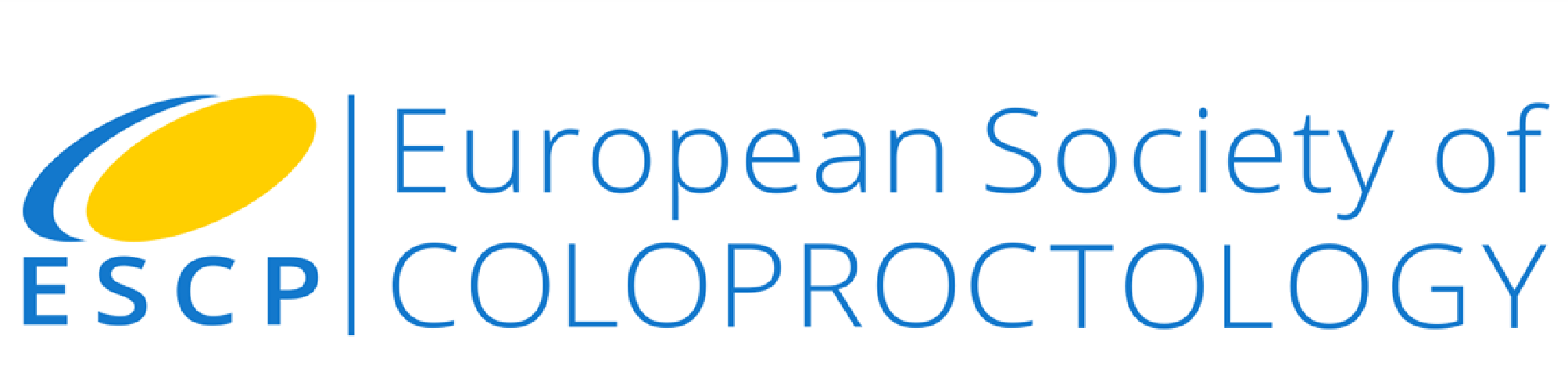


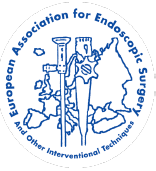

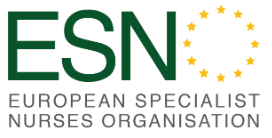

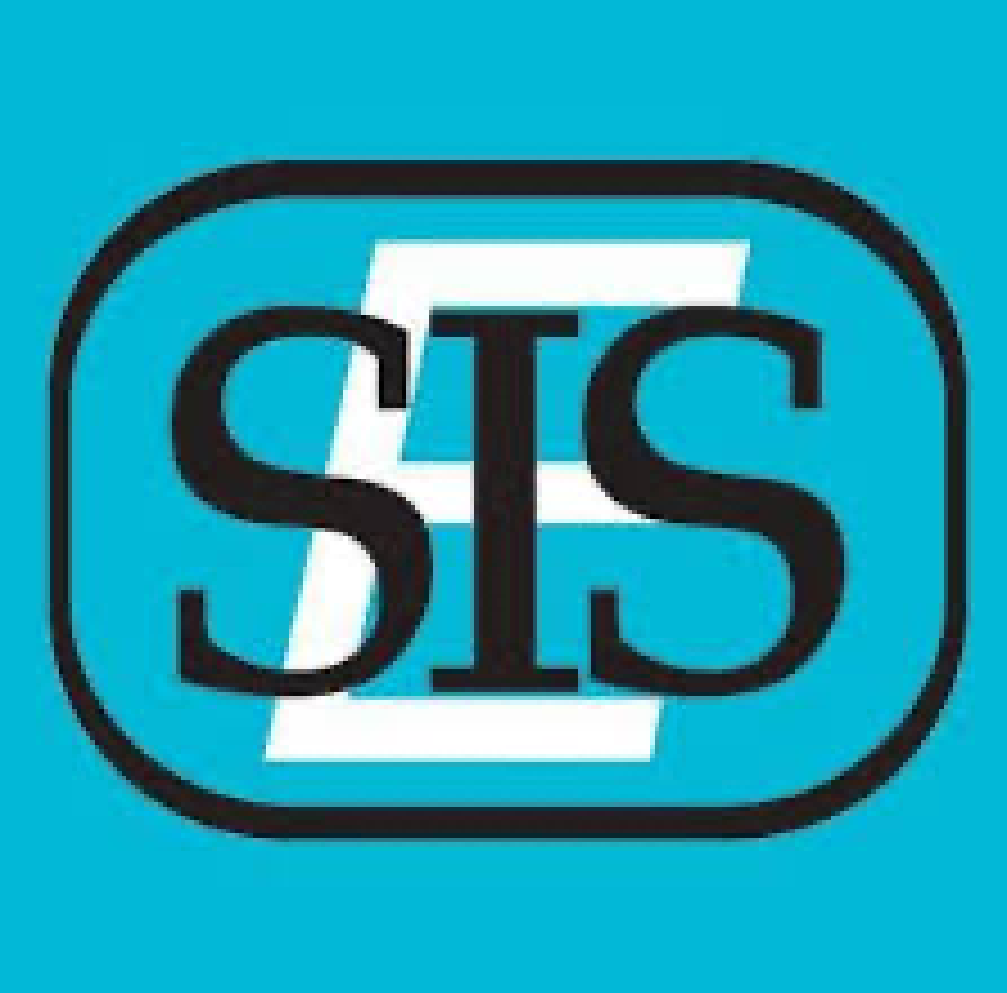


1. **Scope and Purpose**

A surgical site infection (SSI) is a postoperative infection that occurs specifically in a body region where surgery has taken place [1]. SSIs are an important clinical problem, especially in surgical procedures on the digestive tract, which naturally harbours multiple bacteria.

SSIs have a significant impact on patients' reported disability-adjusted life years, as a prolonged stay in hospital and the subsequent complications related to SSI occurrence can have a significant impact on patients’ well-being and quality of life [2,3].

The development of an SSIs (across all surgical specialties) has been reported to cost between €12,167 and €32,000 per patient [4]. European Data from 2004 suggested SSIs were under-reported, with their impact on economic costs 20-years ago ranging from 1.47 and 19.1 billion Euro per year [5,6]. The worldwide risk of developing an SSI after GI surgery is 12.3%, the risk of which can increase to up to nearly 40%, if undergoing contaminated (i.e. infection already present) surgery in a low-income country [7]. SSIs are therefore a substantial financial burden on healthcare systems worldwide.

SSIs can account for over 36% of all hospital acquired infections and are frequently associated with multidrug resistant bacteria [8]. Previously described resistances can be as high as 45% for Staphylococcus aureus and 15% for Escherichia coli [9]. When taking this into account, the economic burden of SSIs can be easier understood, but more concerning is the incidence of multidrug resistant SSIs and the impact that will have in the future.

The purpose of this guideline is to highlight areas where adjustments can be made in practice to reduce the risk of SSI during pre-operative phase. The guideline is intended not only for surgeons but also for healthcare professionals involved in patient preparation, intraoperative management, as well as postoperative and outpatient care. It aims to reduce SSI related disease and economic burden for patients and healthcare systems throughout Europe.

Objectives

This guideline aims to develop evidence-informed recommendations on the prevention of SSI with the objective to reduce the incidence and mitigate the burden of SSI, and improve patient care and experience. We will focus on updating recommendations made by WHO SSI guidelines, focusing specifically on gastrointestinal surgery and pre-operative phase.

**Questions**

The PICO (population, intervention, comparator, and outcome) framework will be used to address the effectiveness and the safety of an intervention and/or a diagnosis method to prevent, treat and assess SSIs. Subgroup analyses considered are comparisons between upper and lower GI surgery, emergency and elective operations and potential patient risk factors for developing SSI (e.g. immunosuppressed, patients with inflammatory bowel disease).

All research questions from the WHO Guidelines to prevent SSI [5] were evaluated to determine if any new evidence was available from 2018 to the present day.

1. Surgical site preparation
2. Preoperative discontinuation of immunosuppressive agents
3. Wound protector
4. Incisional wound irrigation
5. Prophylactic NPWT
6. Antimicrobial prophylaxis with drain
7. Surgical gloves
8. Change of surgical instruments
9. Antimicrobial coated-sutures

| **Topic** | **Research question** | **PICOs** |
| --- | --- | --- |
| Surgical site preparation | *Should chlorhexidine gluconate (CHG) be used compared to povidone-iodine (PVP-I) for patients undergoing GI surgery to prevent surgical site infection?* | **P:** Patients undergoing gastrointestinal, hepatobiliary and pancreatic surgical procedures  **I:** CHG  **C:** PVP-I  **O:** overall SSI rate; superficial SSI rate; deep SSI rate; organ space SSI; overall mortality; quality of life    ***Same PICOs for clean procedures; contaminated procedures; low-income countries; mid-income countries*** |
| Preoperative discontinuation of immunosuppressive agents | *Should immunosuppressive agent(s) be discontinued vs continued for patients undergoing GI surgery?*  *(specifically, steroids and biologics)* | **P:** Patients undergoing gastrointestinal, hepatobiliary and pancreatic surgical procedures.  **I:** continued immunosuppression  **C:** discontinued immunosuppression and timing of discontinuation  **O:** overall SSI rate; superficial SSI rate; deep SSI rate; organ space SSI; overall mortality; quality of life |
| Screening of ESBL | *Should SAP be changed for patients who are undergoing GI surgery and colonised with or a carrier of an extended-spectrum beta-lactamase (ESBL) producing enterobacteriaceae?*    1. Should surgical antibiotic prophylaxis be modified in areas with high (>10%) ESBL-producing Enterobacteriaceae prevalence?  2. Should surgical antibiotic prophylaxis be modified in patients who are known carriers of ESBL-producing Enterobacteriaceae?  3. Should patients be screened for ESBL-producing Enterobacteriaceae prior to surgery? | 1.  **P:** Patients undergoing gastrointestinal, hepatobiliary and pancreatic surgical procedures.  **I:** change of surgical antibiotic prophylaxis in areas with a greater risk of ESBL-producing Enterobacteriaceae  **C:** No change in antibiotic prophylaxis  **O:** overall SSI rate; superficial SSI rate; deep SSI rate; organ space SSI; overall mortality; quality of life    2.  **P:** Patients undergoing gastrointestinal, hepatobiliary and pancreatic surgical procedures.  **I:** Modification of antibiotic prophylaxis for of ESBL-producing Enterobacteriaceae carriers  **C:** No modification of antibiotic prophylaxis for of ESBL-producing Enterobacteriaceae carriers  **O:** overall SSI rate; superficial SSI rate; deep SSI rate; organ space SSI; overall mortality; quality of life    3.  **P:** Patients undergoing gastrointestinal, hepatobiliary and pancreatic surgical procedures.  **I:** routine screening for ESBL-producing Enterobacteriaceae in both low- and high- prevalence areas prior to surgery  **C:** No screening  **O:** overall SSI rate; superficial SSI rate; deep SSI rate; organ space SSI; overall mortality; quality of life |

**Population**

This guideline targets adult patients undergoing gastrointestinal surgery in Europe. We will not have any restriction regarding existing comorbidities and/or type of disease (benign vs malignant for example) but may conduct a sub-analysis stratifying these elements.

**2. Stakeholder involvement**

**Group membership**

This is an international and multi-society collaboration of the European Society of Coloproctology (ESCP), Surgical Infectious Society Europe (SIS-E), European Association of Endoscopic Surgery (EAES), and United European Gastroenterology (UEG), aiming to provide up to date guidance on prevention of SSIs.

A panel of experts from the participating societies has been established as a steering group. The guideline development panel (GDP) will be selected from members of the four European Societies, and will include relevant stakeholders (patient and nursing representatives). The GDG will be balanced according to gender, age, expertise, and geographical location. Each subgroup will comprise a young member with training/experience with guidelines development.

Patients will be invited to join the preliminary meetings, to provide feedback on the protocol, and to vote on choosing relevant outcomes. Involved patients will be asked for their feedback on the manuscripts and will be asked to assist in the writing of a lay summary intended for patients, once the guideline is completed.

**Members of the working group**

**ESCP:**

- Gianluca Pellino, Italy, m <40, colorectal surgeon and lecturer in general surgery, lead applicant on behalf of ESCP, ESCP Guidelines Committee member, Young ESCP chair (LEAD)

- Adele Sayers, UK, f, <40, colorectal surgeon, ESCP Guidelines Committee member (LEAD)

-Yasuko Maeda, UK, f>40, colorectal surgeon, previous ESCP Guidelines Committee member (LEAD)

- Gloria Zaffaroni, Italy, f <40, general surgeon trainee, ESCP Guidelines Committee member, guideline coordinator (YOUNG LEAD)

- Ionut Negoi, Romania, m >40, colorectal surgeon, previous ESCP Guidelines Committee member

- Ruth Blanco-Colino, Spain, f, <40, general surgery trainee, Y-ESCP

- Caterina Foppa, Italy, f, <40, general surgeon, Y-ESCP

- Niki Christou, France, f, <40, colorectal surgeon, ESCP Guidelines Committee member

- Fabian Grass, Switzerland, m, <40, colorectal surgeon, ESCP Guidelines Committee member

**SIS-E:**

- Marja Boermeester, The Netherlands, f >40, intestinal failure and abdominal wall surgery (SIS-E past

president)

- Ines Rubio-Perez, Spain, f <40, colorectal surgeon (SIS-E President Elect)

- Deborah McNamara, Colorectal Surgeon, f >40 Ireland (SIS-E Councillors-at-large)

- Kemal Rasa, Turkey, m>40, HBP surgeon (SIS-E past president)

**EAES:**

- Stavros A Antoniou, Greece, m <40, representative of EAES

- Sheraz Markar, UK, m >40, EAES

- Francesco Maria Carrano, m <40 Italy, EAES

- Monica Ortenzi, f, <40 Italy, EAES

- Daniel Moritz Felsenreich m <40, Austria, EAES

**UEG:**

- Henriette Heinrich, f >40, Switzerland, UEG Education Committee Chair

**European Specialist Nurse Organisation ESNO:**

- Vesna Konjevoda, f, Croatia

**Patient advisory board:**

- Sue Blackwell, f,UK

**Additional members:**

- Jos Kleijnen,UK, m >40, guideline methodologist, retired Professor of Systematic Reviews

- Benito Almirante, Spain, m, >40, infectious disease specialist

- Jean Muris, Netherlands, m>40, Professor of Primary Care, General Practitioner, board member ESPCG

**Target population, preferences and views**

The target population of this guideline is surgeons, anaesthetists, infectious diseases specialists, microbiologists, hospital management, pharmacists, nurses, and general practitioners, involved in the care of patients who undergo digestive surgery.

Patients’ views will be sought from patient representatives with regards to interventions and the weighing of benefits against risks.

**3. Rigour of development**

**Search methods**

A systematic literature review will be conducted to capture relevant papers. PubMed/Medline, EMBASE, Cochrane Central database, Trip (for grey literature) and CINAHL will be interrogated with no language restrictions. Older but relevant literature may be included if deemed necessary. The literature search will be performed with the assistance of clinical information specialists ([www.ksrevidence.com](http://www.ksrevidence.com)) and full search strategies will be added as an appendix.

Given the last update of systematic literature during development of WHO SSI guideline was April 2018, literature published since May 2018 will be the main focus.

Members involved in systematic literature review will screen titles and abstracts relevant to each PICO question. Other members not involved in screening act as referees in case of disagreement.

**Evidence selection criteria**

Evidence will primarily be collated from randomised controlled trials. If there is no RCT, synthesis of data from observational studies will be done if they are deemed suitable to provide evidence following ROBINS-I assessment and exclusion of studies at critical risk of bias (uncontrolled confounders). Sensitivity analysis of studies at low risk of bias will be performed whether downgrading is warranted or not.

Outcomes will be ranked as critical, important and low importance among the panel members, including patients. ESCP, SIS-E, EAES members who are not directly involved in the development process, will be offered an opportunity to comment on the protocol via email invitation and social media.

Some of the outcomes in consideration are: SSI incidence, mortality, quality of life, and serious adverse effects.

Where three or more interventions are available across a network of studies, we will perform network meta-analyses, according to the Cochrane Handbook, using MetaInsight v 4.2.0 tool. [10]

**Strengths and limitations of evidence**

Evidence will be assessed according to GRADE methodology, according to the framework of assessing study design (risk of bias), inconsistency imprecision, indirectness, and publication bias. Data will be collated using GRADEPro. The quality of the evidence will be summarised using the GRADE approach and be classified in four categories: “high”, “moderate”, “low”, and “very low”.

**Formulation of recommendations**

Recommendations will be developed based on GRADE’s Evidence to Decision framework, which will address the quality of evidence, balance between risks and benefits, implementability, use of resources (cost), equity and feasibility, acceptability, and patients’ values and preferences.

Where consensus is required, a Delphi method and ACCORD framework will be used. Agreement will be graded as follows: “strongly agree”, “agree”, “neutral”, “disagree”, “strongly disagree“. Consensus will be defined as an agreement of 80% or over, of all panel members. If no consensus is reached, then all modifications to recommendations will have to agree with GRADE methodology to be considered in the guideline.

**Considerations of benefits and harms**

Both the benefits and the risks of all interventions will be evaluated. Potential trade-off between the benefits of all interventions on SSI prevention and the potential harms will be carefully considered.

**Link between recommendations and evidence**

In addition to the GRADE Evidence to Decision Framework, thought processes by the guideline members leading to recommendations will be made explicit with detailed descriptions.

**External review**

The draft version of the guideline will be posted on a website for consultation with stakeholders and the public interested in this topic.

**Updating procedure**

As new evidence emerged within 5-years from the conception of WHO SSI guideline, review of evidence and update of this guideline will be considered at 5-years from final guideline publication. If little or no new evidence is available at 5-years, this time interval may be extended.

***4. Clarity of presentation***

**Specific and unambiguous recommendations**

Each recommendation will be categorised as ‘strong’ or ‘conditional’ with specific wording used dependent on the quality and strength of evidence. Applicable population and settings will be made clear and caveats to the statement will be explained in detail.

**Management options**

Where different options are applicable, balance of benefits, harms and evidence that could guide choosing a specific intervention will be made clear, taking into account resources (cost) and available infrastructures.

**Identifiable key recommendations**

Key recommendations will be summarised in box as per WHO guidelines and where applicable flow charts will be created.

**5. Applicability**

**Facilitators and barriers to application**

Factors that may influence implementation of this guideline will be sought from a wider audience via public consultation and will be reflected in the implementation strategy.

Multicomponent strategies involving a combination of research (dissemination through email newsletters and social media), ability (providing users with additional resources or tools, such as the interactive guideline tool) and motivational strategies (presentations by key opinion leaders) will be used for dissemination. This approach is likely to encourage implementation, as it will be more effective than using one strategy alone, particularly for guideline adherence.

**Implementation advice/tools**

We will consider producing a snapshot or visual abstract as supplementary material for dissemination and links to algorithms where applicable on the UEG website.

The guideline will be published in UEGJ as an open access article and any other journal that allows publication of the abbreviated version of the guideline. The full guideline will be made available on the websites of ESCP, SIS-E, EAES and UEG, via a link to the UEG-website. Evidence-based prevention and management pathways will be summarised in flow algorithms that will inform the development of a UEG smartphone application.

A dedicated communication strategy will be developed, in collaboration with the Communication Committee of ESCP and the participating societies.

Dedicated educational events and activities (congresses, webinars, courses, interviews) with continued medical education (CME) credits will be organised. Emails, newsletters, social media content and presentations by key opinion leaders will be used for wider dissemination.

**Resource implications**

Health economy will be considered if new or additional intervention suggested by this guideline is likely to incur extra costs.

**Monitoring/auditing criteria**

We will consider local projects to assess implementation of the guideline, and to identify any facilitators/barriers to the use of this guideline.

**6. Editorial independence**

**Funding body**

The guideline work is conducted with UEG Grant but UEG will not influence this guideline as GDP will have full control of the content.

**Competing interests**

One of the steering group members (YM) is the current Guideline Editor for UEGJ. Editorial process is expected to be handled by other editors.

The publications will be authored by the steering group, the guideline panel, and the methodologists on behalf of the Societies with standard or corporate (group) authorship.

**7. Planned timeline**

**Milestone 1:** Working group and outline finalisation

- April – May 2023

**Milestone 2:** GRADE training, outcome grading and PICO questions drafting

- May – July 2023: Finalise protocol
- May – Mid June 2023: JGP and steering committee to draft outcomes and PICO questions
- 15- 30 June: Scoping literature searches with the support of methodologist

**Milestone 3:** Literature search/assessment and recommendations/supporting text drafting

- July - November 2023: Abstract screening by the two independent screeners for each search; retrieval of full-text articles and screening of retrieved literature; grading the evidence.
- December 2023-March 2024: Writing the first draft of the guideline

**Milestone 4:** Delphi exercise (2 rounds) with revision of recommendations as required

- March-April 2024: Consensus and discussion between GDP (comments from those not participating in the working group will be considered, at the discretion of the responsible sub-group)

**Milestone 5:** Finalisation of the recommendations during a consensus meeting, PAB and stakeholders feedback collection

- May 2024: Final consensus meeting (virtual, onsite/real-time voting)
- June 2024: Finalisation of the manuscript/s formatting and language

**Milestone 6:** Manuscript submission

- July 2024: Manuscript submission for publication

**Milestone 7:** Education, implementation, dissemination

- August 2024 onwards: online publication of the guidelines on the collaborating associations websites; social media dissemination; educational courses and activities; presentation at congresses.

**References**

1. <https://www.cdc.gov/hai/ssi/ssi.html>
2. Pinkney TD, Calvert M, Bartlett DC, et al. Impact of wound edge protection devices on surgical site infection after laparotomy: multicentre randomised controlled trial (ROSSINI Trial). BMJ. 2013;347:f4305. Published 2013 Jul 31. doi:10.1136/bmj.f4305
3. Koek MBG, van der Kooi TII, Stigter FCA, de Boer PT, de Gier B, Hopmans TEM, de Greeff SC; Burden of SSI Study Group. Burden of surgical site infections in the Netherlands: cost analyses and disability-adjusted life years. J Hosp Infect. 2019 Nov;103(3):293-302. doi: 10.1016/j.jhin.2019.07.010. Epub 2019 Jul 19. PMID: 31330166.
4. Badia JM, Casey AL, Petrosillo N, Hudson PM, Mitchell SA, Crosby C. Impact of surgical site infection on healthcare costs and patient outcomes: a systematic review in six European countries. J Hosp Infect. 2017 May;96(1):1-15. doi: 10.1016/j.jhin.2017.03.004. Epub 2017 Mar 8. PMID: 28410761.
5. Leaper DJ, van Goor H, Reilly J, Petrosillo N, Geiss HK, Torres AJ, Berger A. Surgical site infection - a European perspective of incidence and economic burden. Int Wound J. 2004 Dec;1(4):247-73.
6. Urban JA. Cost analysis of surgical site infections. Surg Infect (Larchmt). 2006;7 Suppl 1:S19-S22. doi:10.1089/sur.2006.7.s1-19
7. GlobalSurg Collaborative. Surgical site infection after gastrointestinal surgery in high-income, middle-income, and low-income countries: a prospective, international, multicentre cohort study. Lancet Infect Dis. 2018 May;18(5):516-525. doi: 10.1016/S1473-3099(18)30101-4. Epub 2018 Feb 13. PMID: 29452941; PMCID: PMC5910057.
8. Foschi D, Yakushkina A, Cammarata F, Lamperti G, Colombo F, Rimoldi S, Antinori S, Sampietro GM. Surgical site infections caused by multi-drug resistant organisms: a case-control study in general surgery. Updates Surg. 2022 Oct;74(5):1763-1771. doi: 10.1007/s13304-022-01243-3. Epub 2022 Mar 19. PMID: 35304900; PMCID: PMC9481497.
9. Weiner LM, Webb AK, Limbago B, Dudeck MA, Patel J, Kallen AJ, Edwards JR, Sievert DM. Antimicrobial-Resistant Pathogens Associated With Healthcare-Associated Infections: Summary of Data Reported to the National Healthcare Safety Network at the Centers for Disease Control and Prevention, 2011-2014. Infect Control Hosp Epidemiol. 2016 Nov;37(11):1288-1301. doi: 10.1017/ice.2016.174. Epub 2016 Aug 30. PMID: 27573805; PMCID: PMC6857725.
10. <https://training.cochrane.org/handbook/current/chapter-11>
