# Supplementary figures and images for: European Guideline on Pre‐Operative Prevention of Surgical Site Infections Following Digestive Surgery: A Joint Update of the WHO SSI Guideline for Gastrointestinal Surgery by UEG, ESCP, EAES, and SIS‐E
Source: United European Gastroenterol J. 2025 Oct 25;13(10):1887–904. doi: 10.1002/ueg2.70128 (PMC12704574; doi:10.1002/ueg2.70128)

# Appendix 3: PRISMA Flowcharts + risk of bias assessments

##
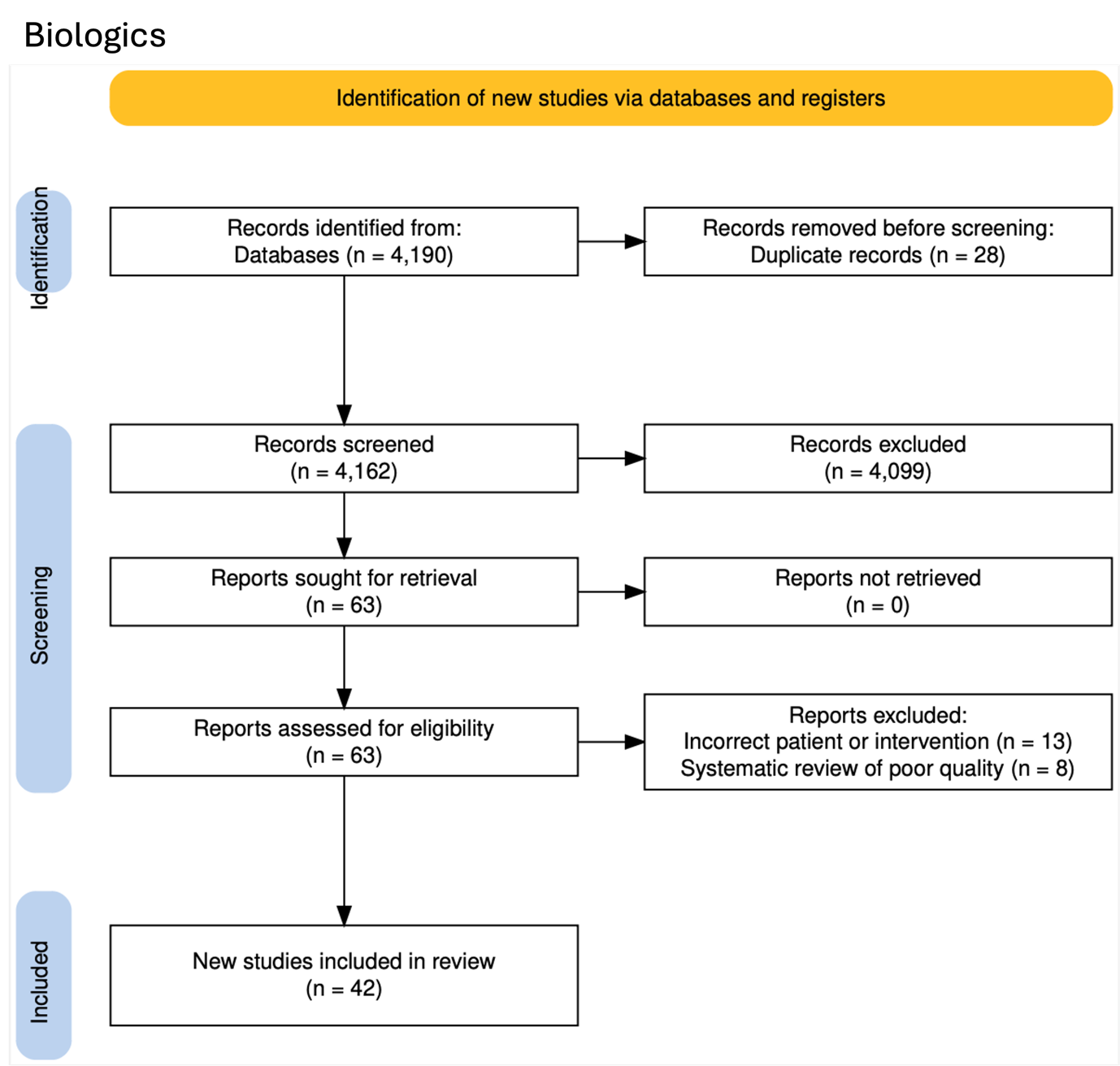


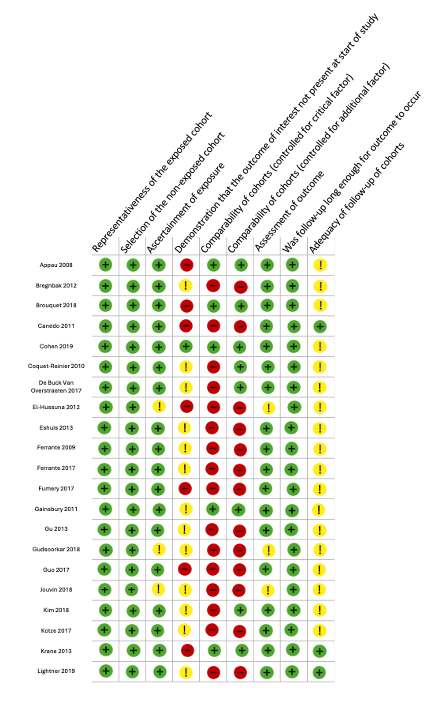


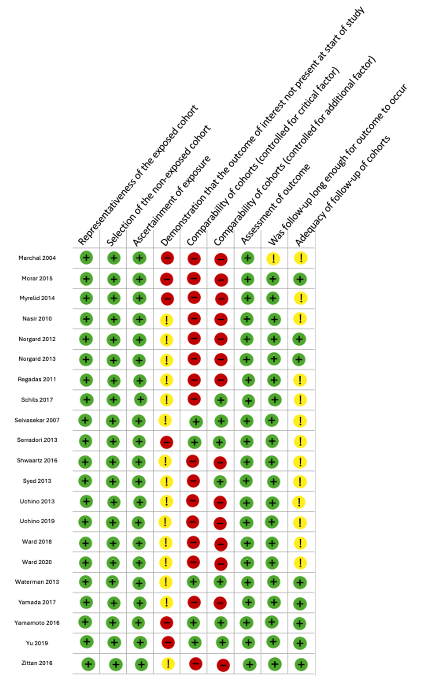


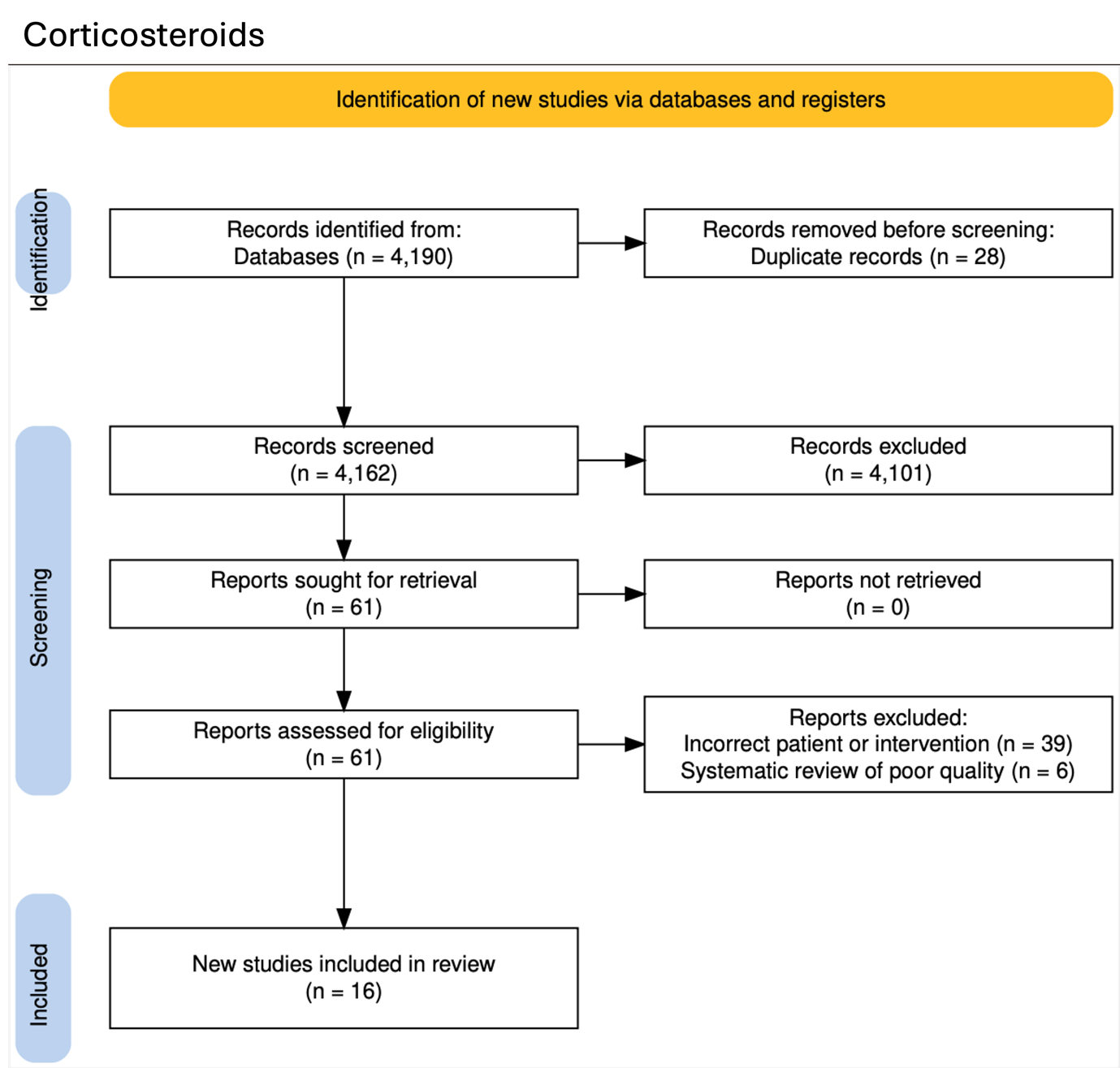


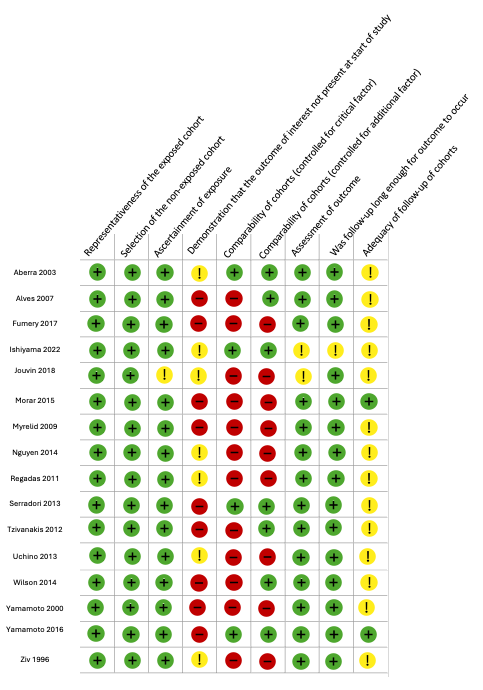


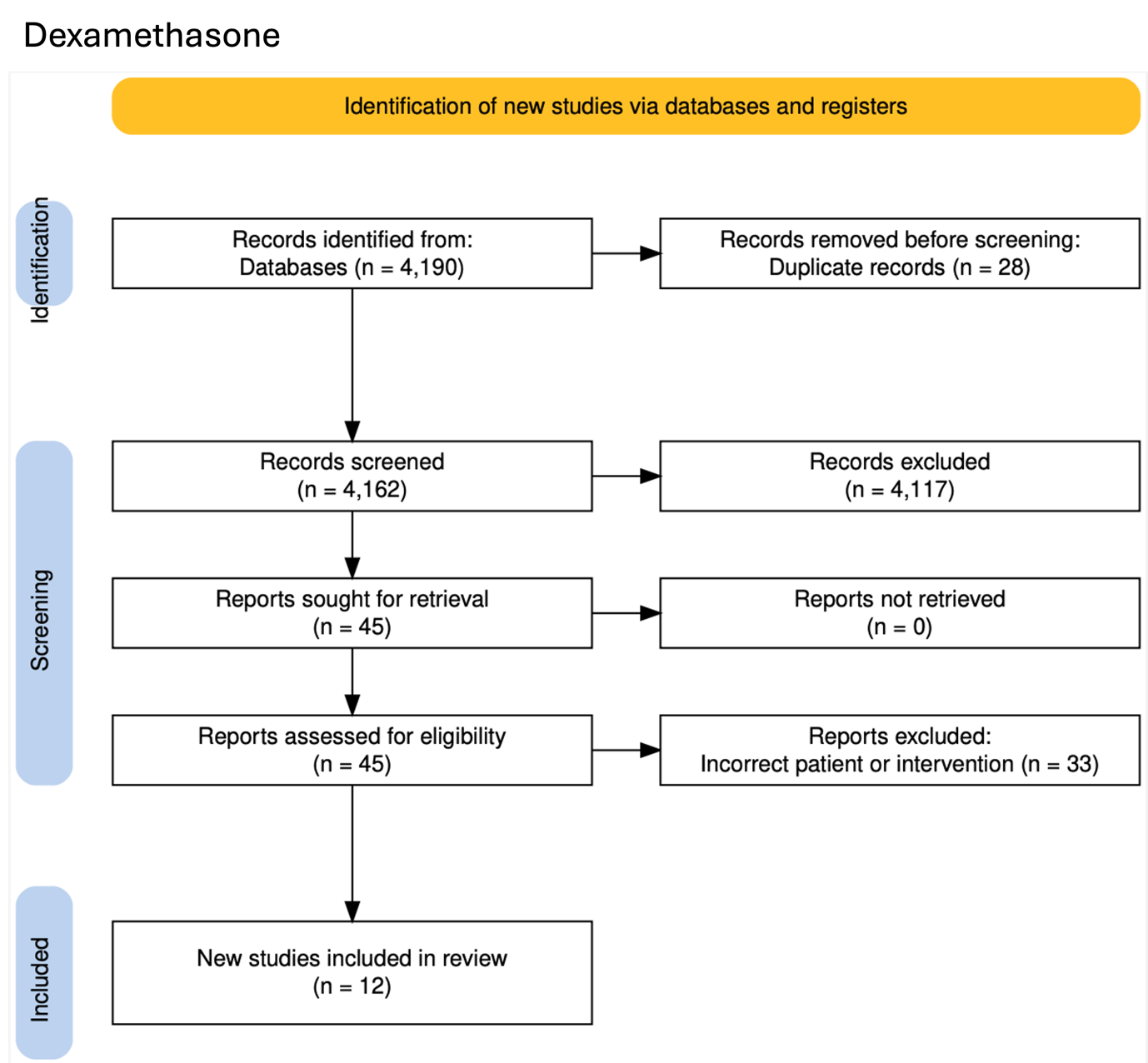


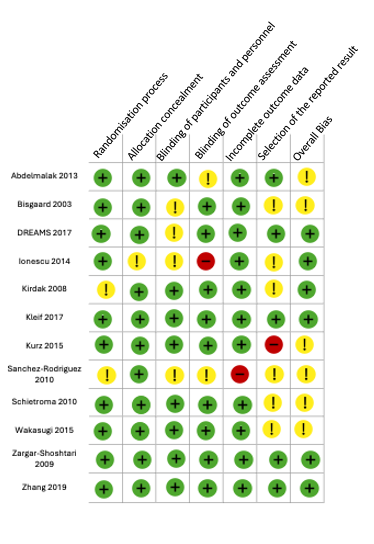


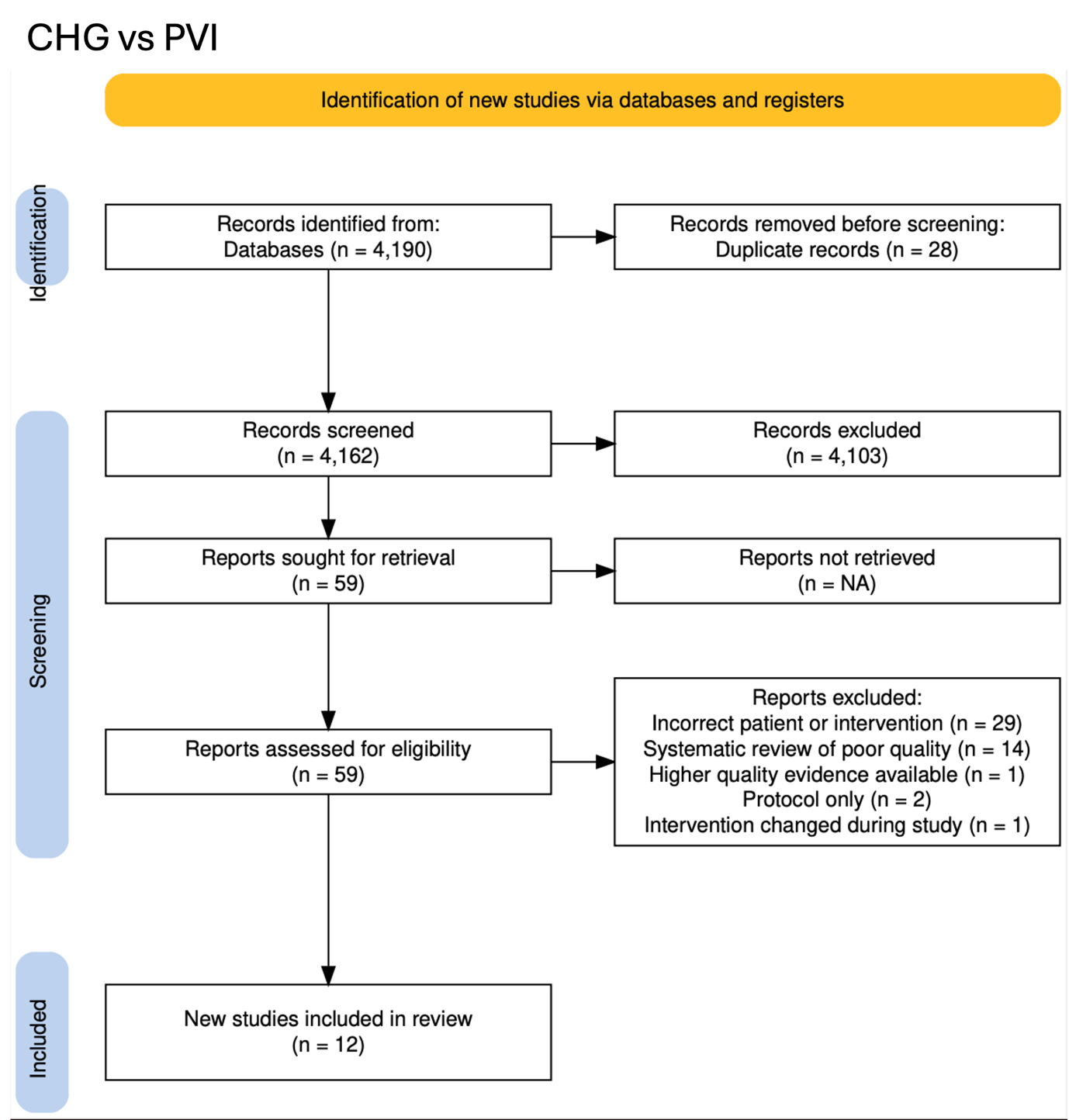


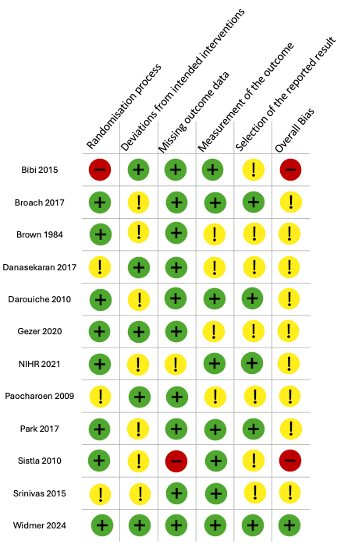


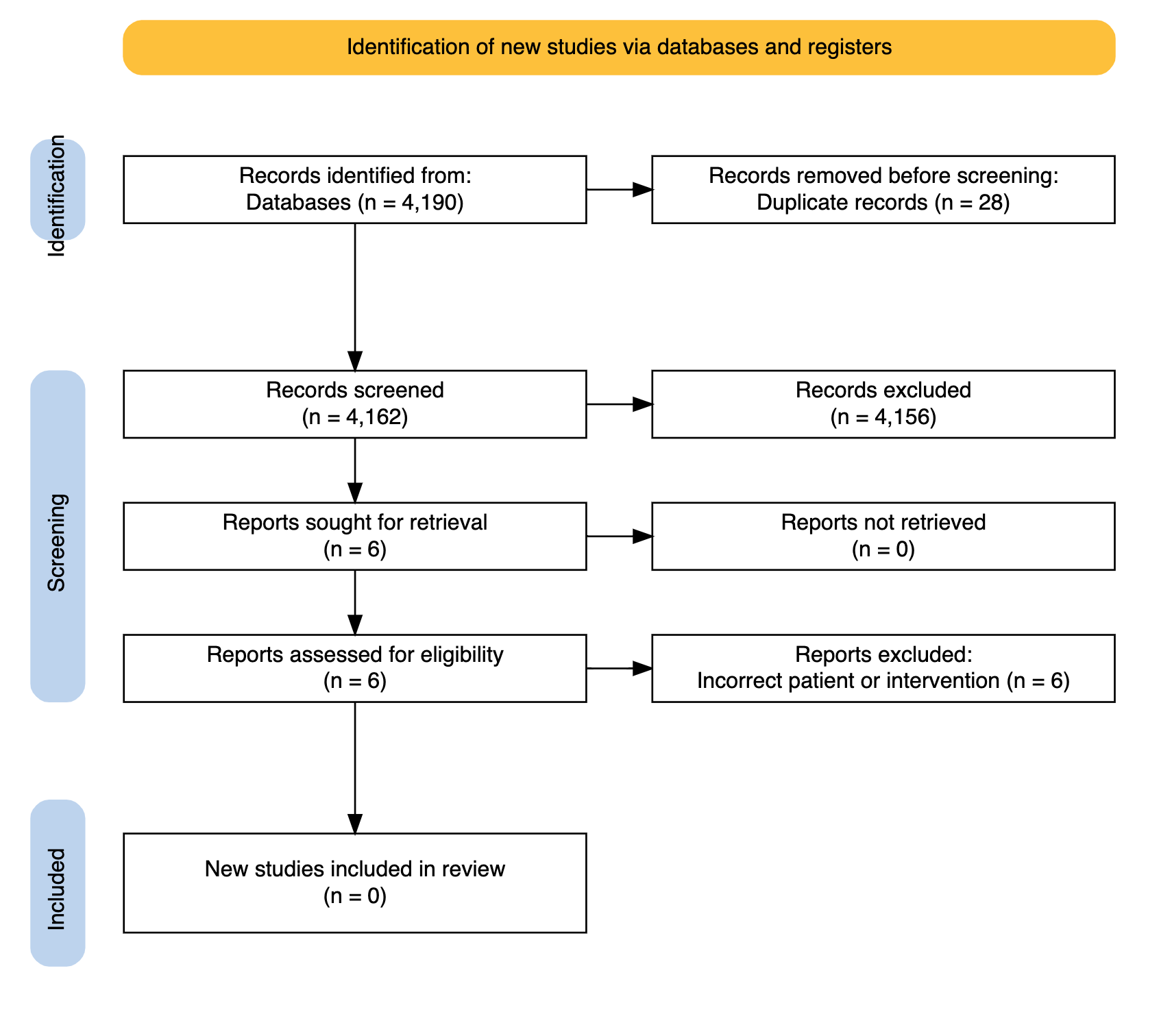


ESBL-producing bacteria

Supplement: Supplementary file 3 — Supporting Information S3 [file UEG2-13-1887-s001.docx]
