# Supplementary material for: European Guideline on Pre‐Operative Prevention of Surgical Site Infections Following Digestive Surgery: A Joint Update of the WHO SSI Guideline for Gastrointestinal Surgery by UEG, ESCP, EAES, and SIS‐E
Source: United European Gastroenterol J. 2025 Oct 25;13(10):1887–904. doi: 10.1002/ueg2.70128 (PMC12704574; doi:10.1002/ueg2.70128)
Supplement: Supplementary file 4 — Supporting Information S4 [file UEG2-13-1887-s002.docx]

# Appendix 4: Forest plots

**Q1 Alcohol-based chlorhexidine gluconate compared to aqueous povidone-iodine**

Overall SSI


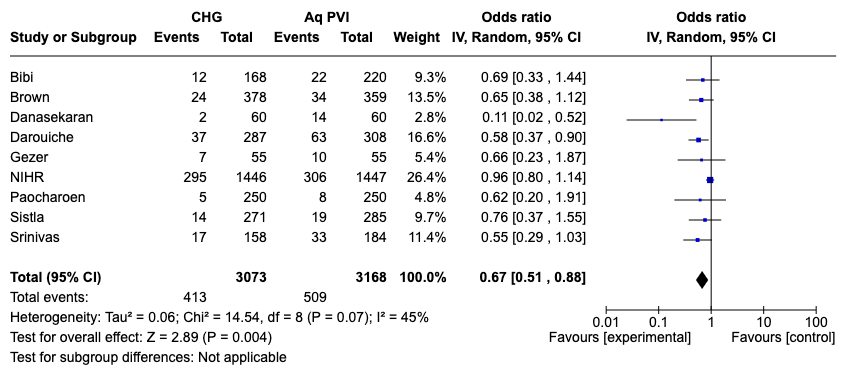


Adverse Events


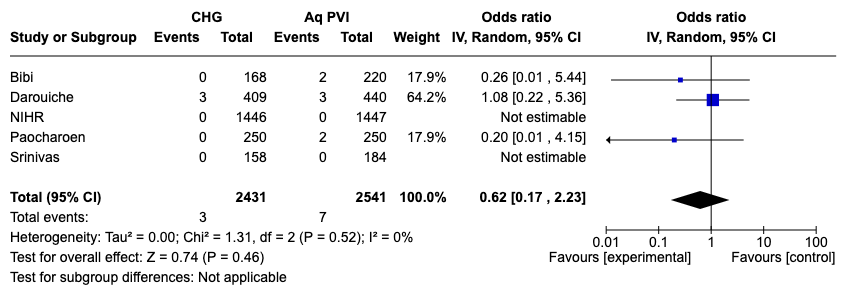


**Q2 Aqueous chlorhexidine gluconate compared to aqueous povidone-iodine**

Superficial SSI


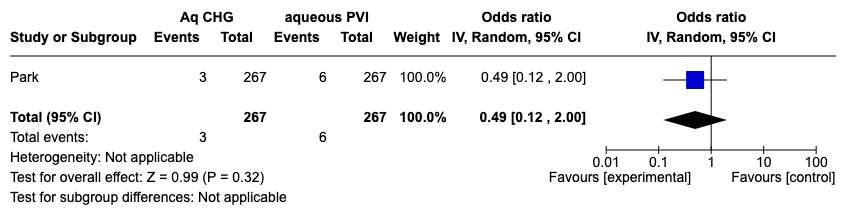


Deep SSI


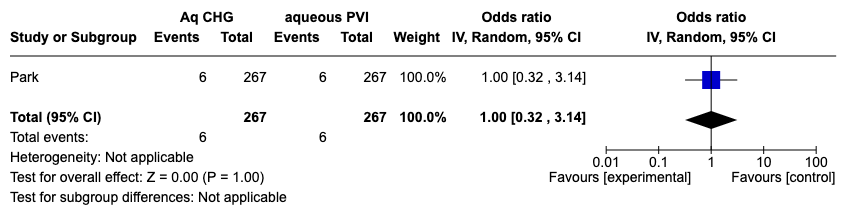


Organ space SSI


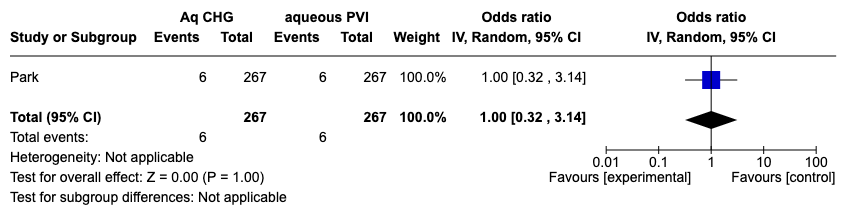


Adverse events


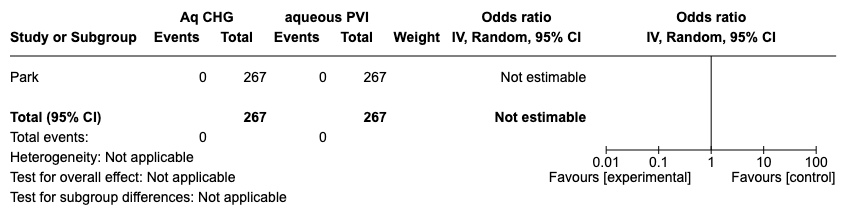


**Q3 Alcohol-based chlorhexidine gluconate 4-5% compared to aqueous povidone-iodine**

Overall SSI


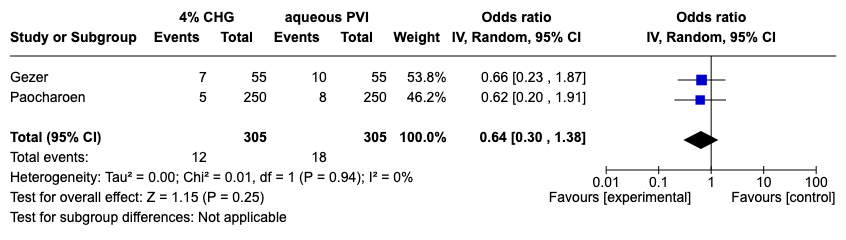


Adverse events


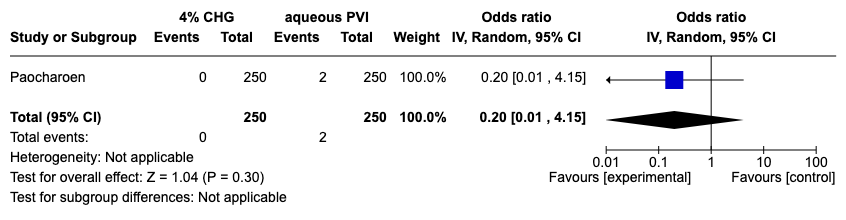


**Q4 Alcohol-based chlorhexidine gluconate 2-3% compared to aqueous povidone-iodine**

Overall SSI

**
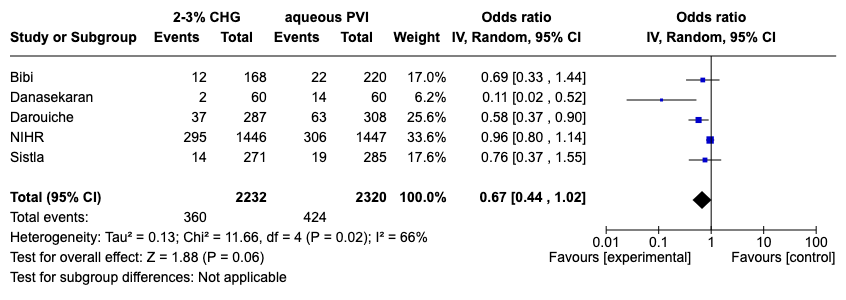
**

Adverse events

**
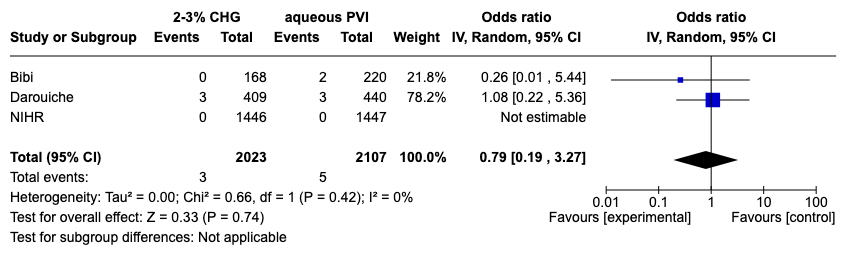
**

**Q5 Alcohol-based chlorhexidine gluconate 2-3% compared to alcohol-based povidone-iodine**

Overall SSI

**
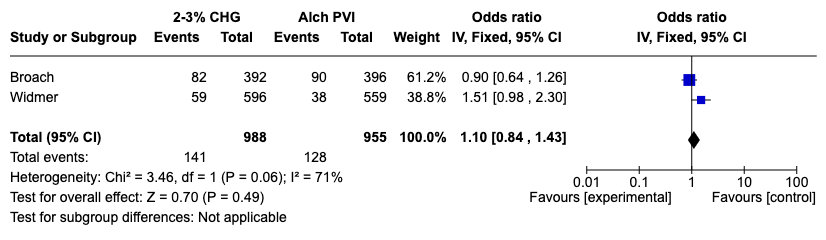
**

Superficial SSI

**
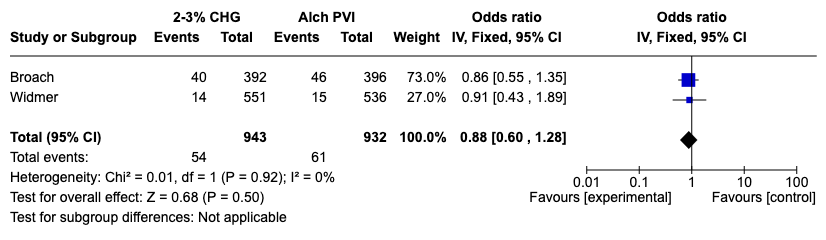
**

Deep SSI

**
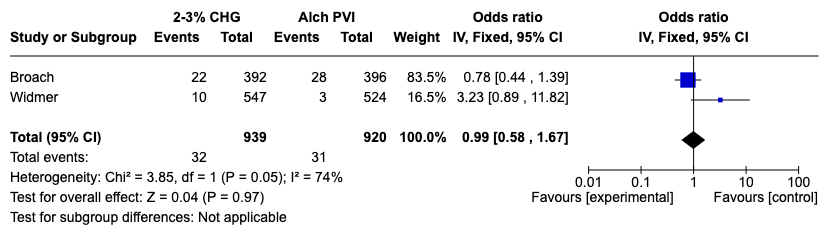
**

Organ space SSI**
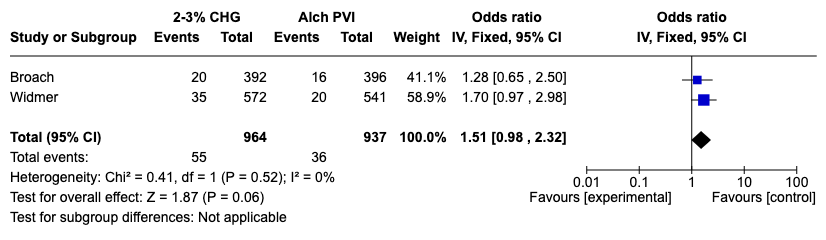
**

Adverse events

**
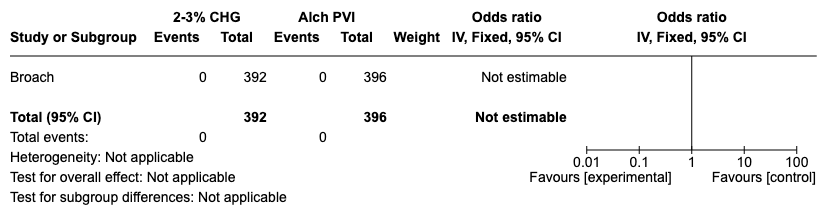
**

**Q6 Preoperative dexamethasone in single-dose vs no preoperative dexamethasone**

Overall SSI

**
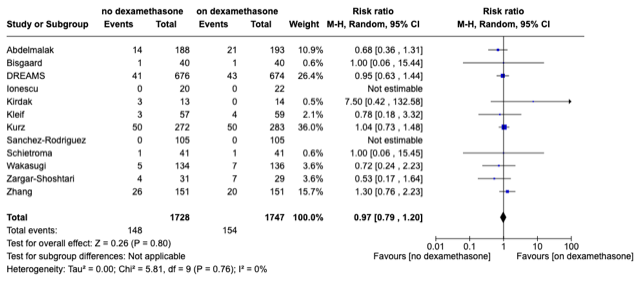
**

**Q7 Discontinuation compared to continuation of corticosteroids**

Overall SSI

**
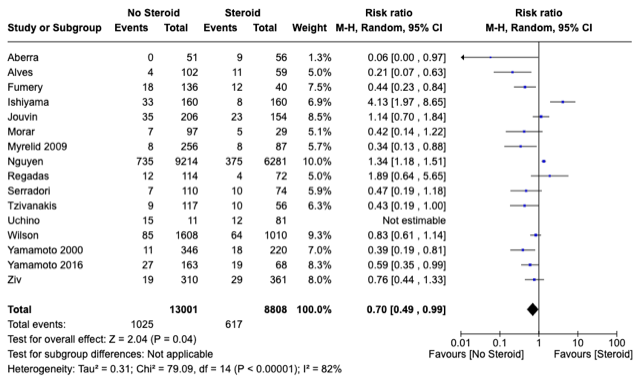
**

Superficial SSI

**
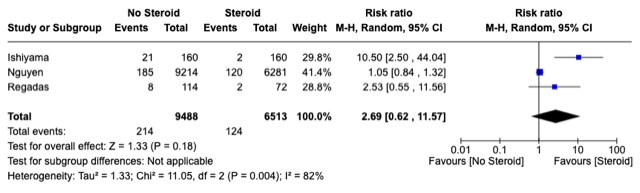
**

Organ space SSI

**
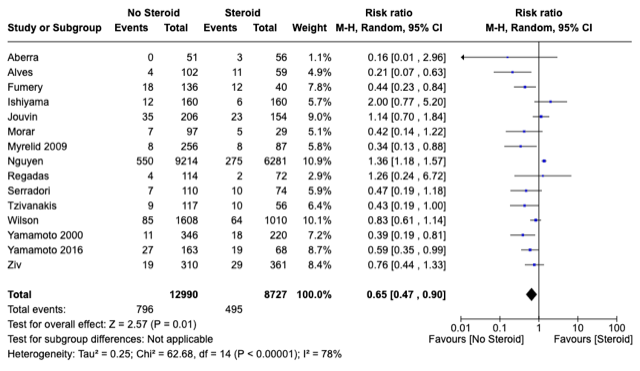
**

**Q8 Discontinuation compared to continuation of anti-TNF**

Overall SSI


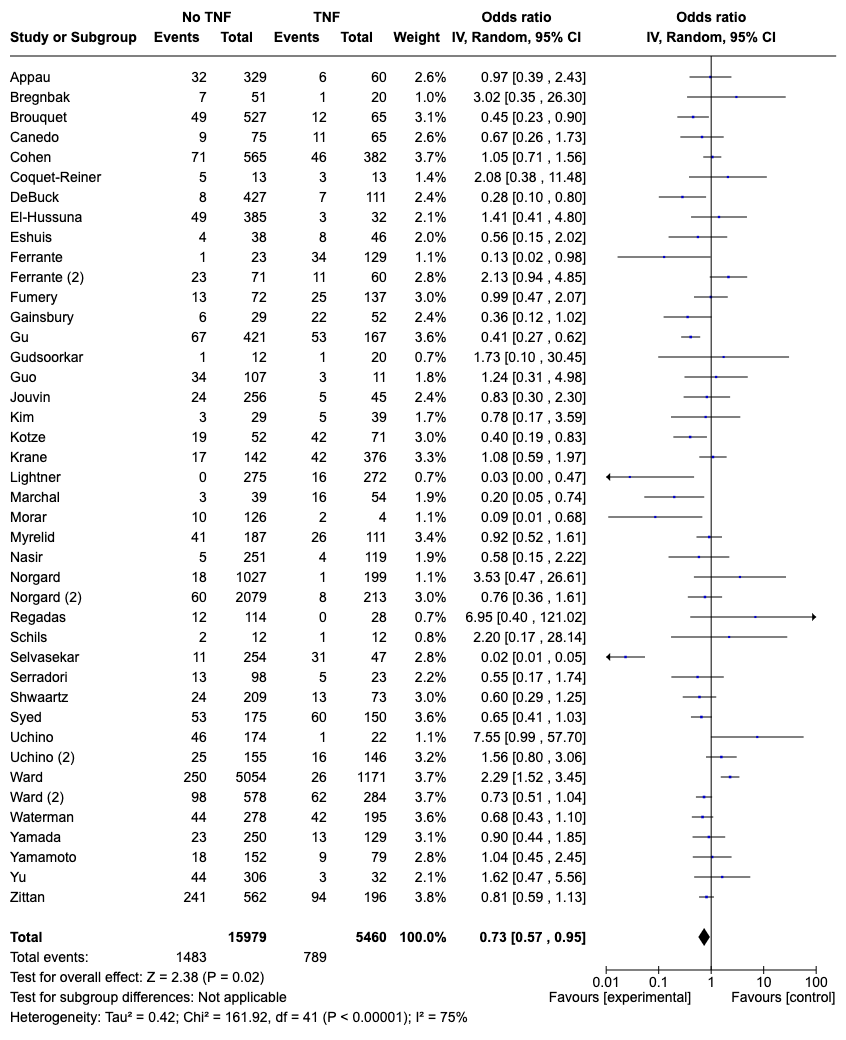


Superficial SSI


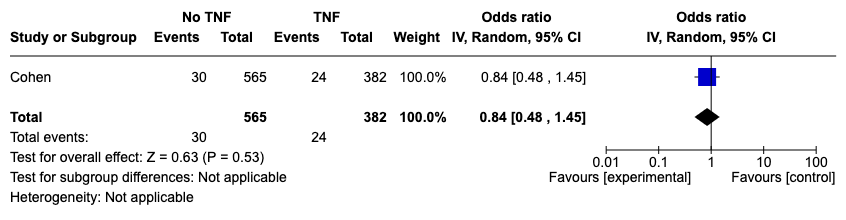


Deep SSI
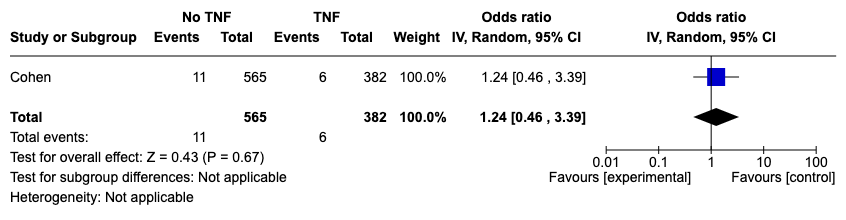


Organ space SSI
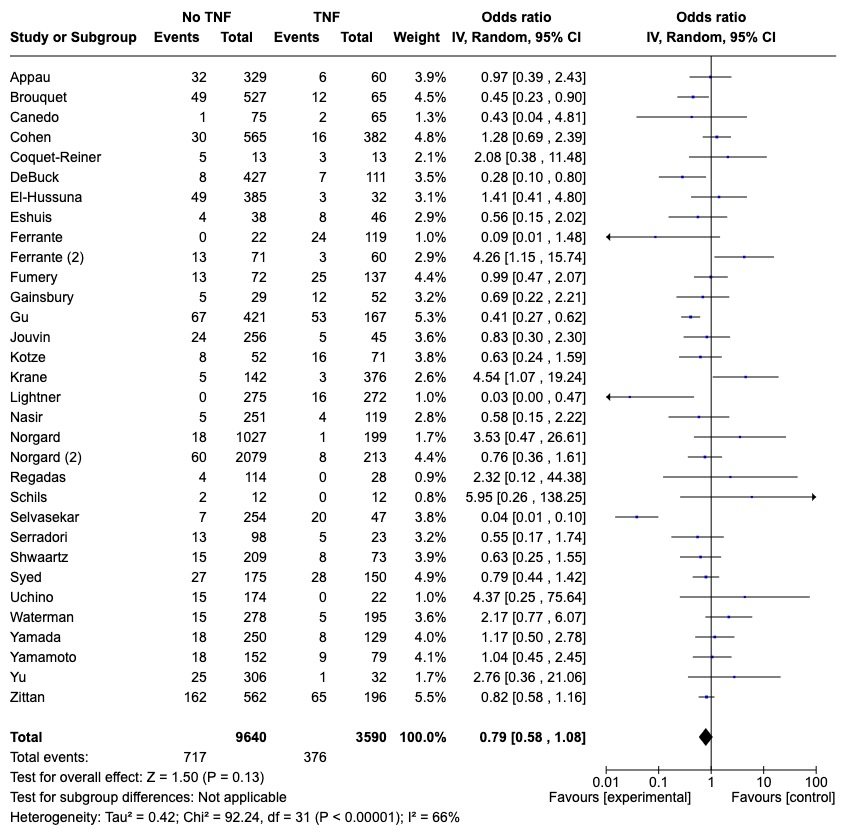


**Q9 Discontinuation compared to continuation of Vedolizumab**

Overall SSI


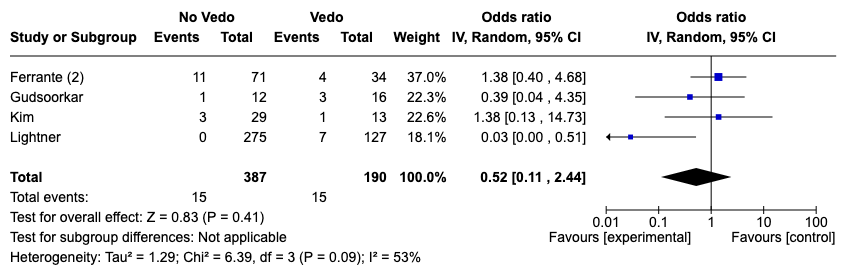


**Q10 Discontinuation compared to continuation of Ustekinumab**

Overall SSI


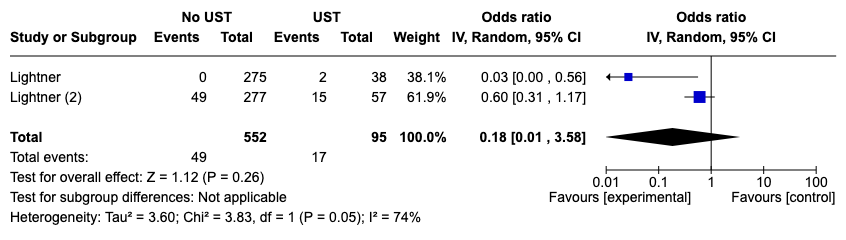


Organ space SSI


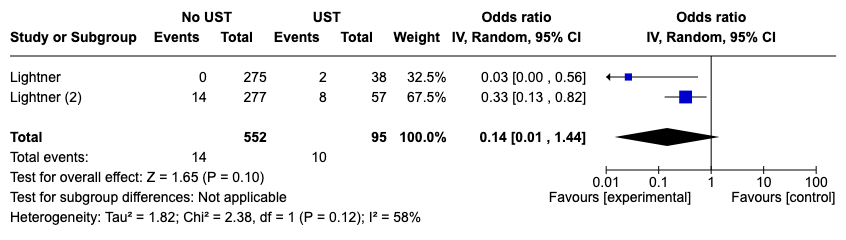


**Q11 Change compared to no change of antibiotic prophylaxis in areas with high (>10%) ESBL-producing Enterobacteriaceae prevalence**

Overall SSI

**
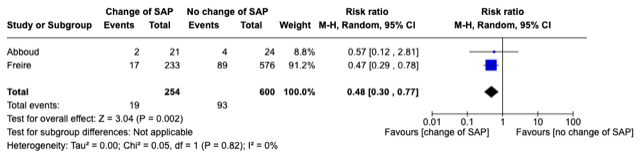
**
